# Lactylation of NAT10 promotes *N*^4^‐acetylcytidine modification on tRNA^Ser-CGA-1-1^ to boost oncogenic DNA virus KSHV reactivation

**DOI:** 10.1038/s41418-024-01327-0

**Published:** 2024-06-15

**Authors:** Qin Yan, Jing Zhou, Yang Gu, Wenjing Huang, Mingpeng Ruan, Haoran Zhang, Tianjiao Wang, Pengjun Wei, Guochun Chen, Wan Li, Chun Lu

**Affiliations:** 1 Department of Microbiology, Nanjing Medical University, Nanjing, 211166 PR China; 2 Key Laboratory of Pathogen Biology of Jiangsu Province, Nanjing Medical University, Nanjing, 211166 PR China; 3 Changzhou Medical Center, Nanjing Medical University, Nanjing, 211166 PR China; 4 Department of Infectious Diseases, Changzhou Third People’s Hospital, Changzhou, 213000 PR China

**Keywords:** Microbiology, Infectious diseases

## Abstract

*N*^4^-acetylcytidine (ac^4^C), a conserved but recently rediscovered RNA modification on tRNAs, rRNAs and mRNAs, is catalyzed by *N*-acetyltransferase 10 (NAT10). Lysine acylation is a ubiquitous protein modification that controls protein functions. Our latest study demonstrates a NAT10-dependent ac^4^C modification, which occurs on the polyadenylated nuclear RNA (PAN) encoded by oncogenic DNA virus Kaposi’s sarcoma-associated herpesvirus (KSHV), can induce KSHV reactivation from latency and activate inflammasome. However, it remains unclear whether a novel lysine acylation occurs in NAT10 during KSHV reactivation and how this acylation of NAT10 regulates tRNAs ac^4^C modification. Here, we showed that NAT10 was lactylated by α-tubulin acetyltransferase 1 (ATAT1), as a writer at the critical domain, to exert RNA acetyltransferase function and thus increase the ac^4^C level of tRNA^Ser-CGA-1-1^. Mutagenesis at the ac^4^C site in tRNA^Ser-CGA-1-1^ inhibited its ac^4^C modifications, translation efficiency of viral lytic genes, and virion production. Mechanistically, KSHV PAN orchestrated NAT10 and ATAT1 to enhance NAT10 lactylation, resulting in tRNA^Ser-CGA-1-1^ ac^4^C modification, eventually boosting KSHV reactivation. Our findings reveal a novel post-translational modification in NAT10, as well as expand the understanding about tRNA-related ac^4^C modification during KSHV replication, which may be exploited to design therapeutic strategies for KSHV-related diseases.

## Introduction

Post-transcriptional modifications on RNAs can be mapped to discover how they regulate gene expression via messenger RNAs (mRNAs), transfer RNAs (tRNAs), ribosomal RNAs (rRNAs) and other non-coding RNAs. Currently, more than 170 types of chemical RNA modifications have been discovered [1]. As the first acetylation mark in RNA, *N*^4^-acetylcytidine (ac^4^C) at the fourth position of cytidine (C) bases in RNAs is regarded as a conserved nucleoside in eukaryotes and prokaryotes [2]. Most of the ac^4^C studies focus on the observations from tRNAs and rRNAs, but recently has also been detected in human and yeast mRNAs [3–5]. In all cases, ac^4^C production is catalyzed by the *N*-acetyltransferase 10 (NAT10) or its homologs [5], while NAT10 is the only human enzyme known to have both acetyltransferase and RNA-binding activities [3].

In addition to RNA post-transcriptional modifications, protein post-translational modifications (PTMs) have also been widely recognized to act in cellular homeostasis and disease progression [6]. Except for well-characterized PTMs (e.g., acetylation and ubiquitination), an array of novel post-translational modifications have been elucidated, such as lysine propionylation (Kpr), 2-hydroxyisobutyrylation (Khib) and lactylation (Kla), which are dynamic, reversible, and modulated by specific enzymes that can add or remove functional groups [7]. Notably, lactate-derived lysine residue lactylation, occurring on both histone and non-histone proteins, participates in various physiological and pathological processes [8, 9]. Although NAT10 was identified as a substrate for Khib modification to increase its stability in promoting cancer metastasis [10], little is known about the other post-translational modifications on NAT10, such as lactylation.

Emerging studies have shown that aberrant NAT10-dependent RNA ac^4^C modification is associated with viral infection. For instance, ac^4^C modification appears in the genomes of RNA viruses, including HIV-1, enterovirus 71 and influenza A virus, and undertakes critical roles in viral RNA stability and replication [11–13]. Interestingly, our latest study has also identified that during infection, ac^4^C modification is realized by oncogenic DNA virus Kaposi’s sarcoma-associated herpesvirus (KSHV) [14]. KSHV is the causative agent of Kaposi’s sarcoma (KS), primary effusion lymphoma (PEL), multicentric Castleman’s diseases (MCD), and KSHV-associated inflammatory cytokine syndrome (KICS) [15, 16]. Like other herpesviruses, KSHV exhibits a biphasic life cycle: latent and lytic, with distinct expression profiles [17]. During the latent phase, KSHV genome exists as a circular episome in the infected cells, with a restricted expression. Upon switch to the lytic phase, three classes of lytic genes are expressed in a temporally regulated manner, namely immediate early (IE), early (E), and late (L), resulting in the assembly and release of progeny virions [18]. Both phases may be regulated to control KSHV-induced tumorigenesis and viral propagation. Therefore, delving into regulatory mechanisms of KSHV life cycle may help to design therapeutic strategies for viral infections and related diseases.

Through genome-wide analysis of KSHV transcripts identified at NAT10-specific ac^4^C sites, we have discovered that NAT10 catalyzes ac^4^C into the polyadenylated nuclear RNA (PAN), a long non-coding RNA encoded by KSHV, to stabilize PAN and facilitates viral reactivation. Meanwhile, PAN ac^4^C modification also eases the recruitment of IFN-γ-inducible protein-16 (IFI16) mRNA by NAT10, to strengthen its effect on ac^4^C acetylation, mRNA stabilization and inflammasome activation [14]. Given that tRNAs are the most densely modified by NAT10 [5, 19, 20], we speculated that the tRNAs of KSHV-encoded lytic genes might also employ NAT10-mediated ac^4^C modification to increase translation efficiency and promote viral reactivation.

In this work, we demonstrated that acetyltransferase NAT10 is lactylated at Lys290 by α-tubulin acetyltransferase 1 (ATAT1) that adds acyl-lysine modifications, thus inducing ac^4^C modification on tRNA^Ser-CGA-1-1^ to promote KSHV lytic transcripts translation and reactivation. Mechanically, KSHV PAN enhances the interaction between NAT10 and ATAT1, which in turn promotes NAT10 lactylation to boost KSHV reactivation. Together, our findings clarified the cooperation of lysine lactylation modifications with RNA ac^4^C modifications during KSHV infection, and the action of ac^4^C modification on tRNA viral translation in KSHV life cycle. ac^4^C may serve as a potential therapeutic target for viral infectious diseases.

## Results

### NAT10-dependent ac^4^C modification on tRNA^Ser-CGA-1-1^ promotes KSHV lytic genes translation and virion production

**Fig. 1:**
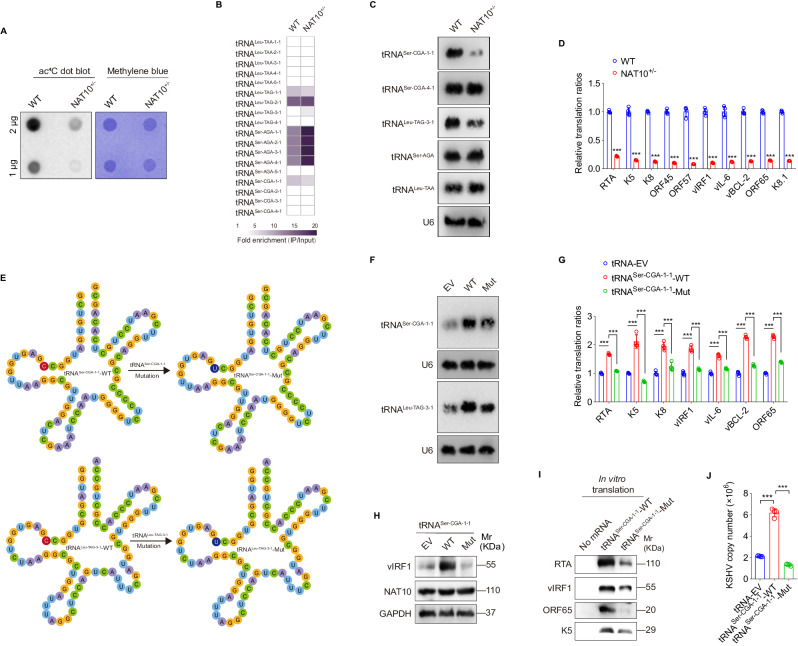
NAT10 catalyzes tRNA^Ser-CGA-1-1^ ac^4^C modification to promote viral mRNAs translation and virion production. **A** The total RNAs from iSLK-KSHV NAT10^+/-^ cells (NAT10^+/-^) and iSLK-KSHV NAT10^+/+^ cells (WT) were subjected to dot blot assay with anti-ac^4^C antibody. Methylene blue staining was used as the internal control. **B** The heatmap for differentially expressed tRNAs in cells shown as in (**A**) by tRNA acRIP-seq analysis. The pseudo-color represented as the fold enrichment (IP/Input) of WT and NAT10^+/−^ group, respectively. Any tRNAs not detected in tRNA acRIP-seq were plotted as white in the heatmap. **C** The total RNAs from cells shown as in (**A**) were subjected to Northern blot with probes to tRNA^Ser-CGA-1-1^, tRNA^Ser-CGA-4-1^, tRNA^Leu-TAG-3-1^, tRNA^Ser-AGA^, tRNA^Leu-TAA^, and U6 as the control. **D** The RT-qPCR analysis for the ribosome-nascent chain-complex-bound mRNA (RNC-qPCR) of representative viral genes of KSHV (RTA, K5, K8, ORF45, ORF57, vIRF1, vIL-6, vBCL-2, ORF65, and K8.1) in cells shown as in (**A**). ***P < 0.001 by Student’s *t*-test. **E** Schematic representation of the acetylation site mutation of tRNA^Ser-CGA-1-1^ (Upper) and tRNA^Leu-TAG-3-1^ (Lower). The C (marked in red) at position 12 was mutated to U (marked in dark blue). **F** The total RNAs from iSLK-KSHV cells with overexpression of the wild type tRNA^Ser-CGA-1-1^ or tRNA^Leu-TAG-3-1^ (WT), mutant tRNA^Ser-CGA-1-1^ or tRNA^Leu-TAG-3-1^ (Mut), and their control empty vector (**EV**) for 48 h were respectively subjected to Northern blot with probes to tRNA^Ser-CGA-1-1^, tRNA^Leu-TAG-3-1^ and U6 as the control. **G**. The RT-qPCR analysis for the ribosome-nascent chain-complex-bound mRNA (RNC-qPCR) of representative viral genes of KSHV (RTA, K5, K8, vIRF1, vIL-6, vBCL-2, and ORF65) in iSLK-KSHV cells with overexpression of the wild type (tRNA^Ser-CGA-1-1^-WT) or its mutant (tRNA^Ser-CGA-1-1^-Mut) tRNA^Ser-CGA-1-1^ for 48 h, and their control (tRNA-EV). ***P < 0.001 by Student’s *t*-test. **H** The expression levels of vIRF1 and NAT10 in iSLK-KSHV cells with overexpression of the wild type (WT), mutant (Mut) tRNA^Ser-CGA-1-1^, and their control (EV) for 48 h were detected by Western blot. **I** The SDS gel analysis of the in vitro translation reaction supplemented with the wild type (tRNA^Ser-CGA-1-1^-WT) or the mutant (tRNA^Ser-CGA-1-1^-Mut) tRNA^Ser-CGA-1-1^, in comparison with reaction mixtures that not containing mRNA (No mRNA). **J** By the assessment of ORF26, real-time DNA-PCR for cells shown as in (**H**) was performed to detect viral copy number after doxycycline stimulation for 72 h. ***P < 0.001 by Student’s *t*-test.

To investigate whether NAT10 promotes KSHV lytic reactivation via ac^4^C modification on tRNAs other than PAN as a viral long non-coding RNA [14], the NAT10-knockdown iSLK-KSHV cells (NAT10^+/-^) were generated by CRISPR-Cas9-based gene editing in the iSLK-KSHV system [14], in which KSHV reactivation can be triggered by doxycycline and sodium butyrate [21]. Anti-ac^4^C based dot blot showed that the ac^4^C levels decreased significantly in the total RNA of NAT10-knockdown iSLK-KSHV cells (Fig. 1A). tRNA^Ser-CGA-1-1^, tRNA^Leu-TAG-3-1^ and tRNA^Ser-CGA-4-1^ are the three known ac^4^C-modified tRNAs identified by a chemical genomic method [4]. tRNA acRIP-seq with ac^4^C antibody demonstrated that NAT10 knockdown altered the ac^4^C modification levels in tRNA^Ser-CGA-1-1^, but not in tRNA^Leu-TAG-3-1^ and tRNA^Ser-CGA-4-1^ (Fig. 1B). Furthermore, NAT10 knockdown decreased the expression levels of tRNA^Ser-CGA-1-1^ and tRNA^Leu-TAG-3-1^, but had no effect on those of tRNA^Ser-CGA-4-1^, tRNA^Ser-AGA^ and tRNA^Leu-TAA^ as the non-ac^4^C control (Fig. 1C).

Given that tRNAs are essential modulators for mRNA translation, quantitative PCR of ribosome-nascent chain-complex-bound mRNA (RNC-qPCR) was performed to evaluate the activity of viral lytic tRNAs upon NAT10 knockdown. Surprisingly, NAT10 knockdown significantly reduced the translation ratios (TRs) of a series of viral lytic transcripts, including immediate early genes (RTA, K5, K8 and ORF45), early genes (ORF57, vIRF1, vIL-6 and vBCL-2) and late genes (ORF65 and K8.1) (Fig. 1D). To explore whether NAT10 regulates viral mRNAs translation via tRNA ac^4^C modification, the acetylation sites at position 12 in tRNA^Ser-CGA-1-1^ and tRNA^Leu-TAG-3-1^ were mutated from C to U (Fig. 1E), but the expression levels of mutants did not change much in general (Fig. 1F). RNC-qPCRs revealed that only ac^4^C-modified tRNA^Ser-CGA-1-1^, rather than non-ac^4^C tRNA^Ser-CGA-1-1^ and tRNA^Leu-TAG-3-1^, increased the translation ratios of KSHV lytic transcripts (Fig. 1G and S1). Furthermore, overexpression of tRNA^Ser-CGA-1-1^, but not its ac^4^C site mutant, increased the protein abundance of vIRF1 (Fig. 1H). To ensure the effect of ac^4^C-modified tRNA^Ser-CGA-1-1^ on viral mRNAs translation, a cell-free translation system was utilized to omit tRNAs from the translation mix, with the addition of the wild-type tRNA^Ser-CGA-1-1^ or its ac^4^C site mutant. Consistent with the results in vivo, mutating the ac^4^C site of tRNA^Ser-CGA-1-1^ inhibited the synthesis of KSHV RTA, vIRF1, ORF65 and K5 (Fig. 1I). Consequently, the KSHV genome copy numbers also increased significantly upon tRNA^Ser-CGA-1-1^ overexpression, while non-ac^4^C tRNA^Ser-CGA-1-1^ achieved an opposite effect (Fig. 1J). These data collectively suggest that NAT10-mediated ac^4^C modification on tRNA^Ser-CGA-1-1^ enhances the translation of KSHV lytic transcripts and virion production.

### NAT10 lactylation at Lys290 facilitates KSHV lytic genes translation and virion production

**Fig. 2:**
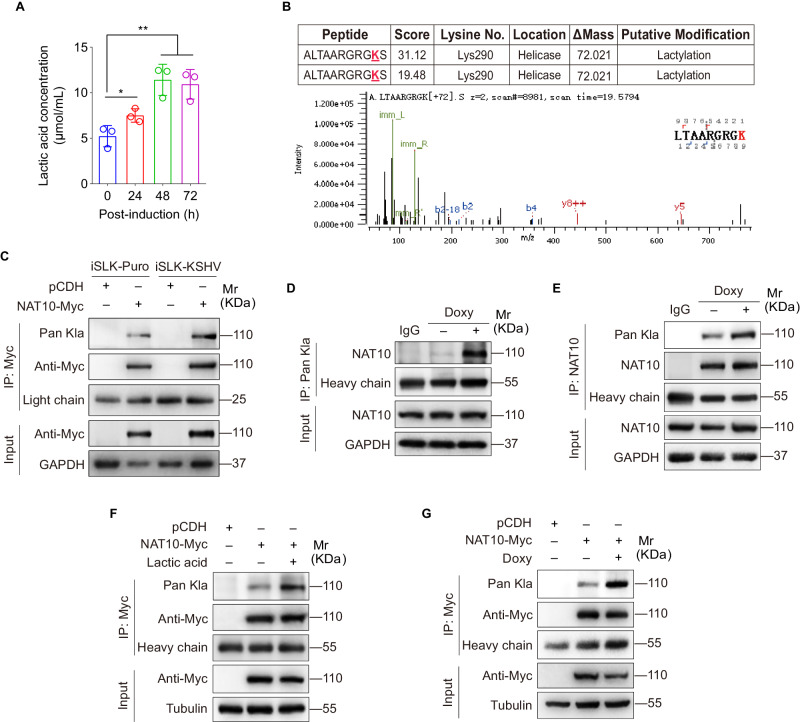
NAT10 undergoes lactate-driven lactylation during KSHV reactivation. **A** The culture medium of iSLK-KSHV cells with doxycycline induction for 0, 24, 48, and 72 h were harvested for detection of lactic acid concentration with the Lactate Assay Kit (Jiancheng BioEngineering, Nanjing, China). *P < 0.05 and **P < 0.01 by Student’s *t*-test versus the 0 h group. **B** Lactylation of NAT10 lysine residues in iSLK-KSHV cells transduced with lentiviral NAT10 was identified by LC–MS/MS analysis. The sequence of identified peptides, score of peptide segment for matching degree, the number and location of the identified lysines (**K**) in LC-MS/MS analysis were shown (Upper). The increased 72.021 Da on lysines was considered as lactylation. The b and y ions in the spectra of the peptide were marked in blue and red, respectively (Lower). **C** The iSLK-Puro or iSLK-KSHV cells were transduced with lentiviral NAT10 (NAT10-Myc) or control lentivirus (pCDH) for 48 h, and then subjected to anti-Myc immunoprecipitation assay (IP). Lysine lactylation in the immunoprecipitated NAT10 was examined by Western blot with anti-Pan-lactyl-lysine antibody (Pan Kla). **D**, **E** The iSLK-KSHV cells induced with (+) or without (−) doxycycline (Doxy) were subjected to the anti-Pan Kla (**D**) or anti-NAT10 (**E**) immunoprecipitation, and the pull-downed proteins were examined by Western blot with anti-NAT10 (**D**) or anti-Pan Kla (**E**) antibodies, respectively. Anti-IgG antibody was used as the control. **F** The iSLK-KSHV cells transduced with lentiviral NAT10 (NAT10-Myc) or control lentivirus (pCDH) for 48 h were treated with or without lactic acid (20 mM) for 24 h, and then subjected to anti-Myc IP assay and detected by Western blot with the anti-Pan Kla antibody. **G** The iSLK-KSHV cells transduced with lentiviral NAT10 (NAT10-Myc) or control lentivirus (pCDH) for 48 h were treated with or without doxycycline (Doxy) for 72 h, and then subjected to anti-Myc IP assay and detected by Western blot with the anti-Pan Kla antibody.

Our latest study has demonstrated that KSHV reactivation does not affect the expression level of NAT10 protein [14], but the mechanisms underlying NAT10-mediated KSHV reactivation need further explorations. We and others have previously found that lactate accumulates not only during KSHV latent infection [22, 23], but also in the medium of iSLK-KSHV cells after doxycycline induction (Fig. 2A), indicating that lactate-driven lysine lactylation may be involved in NAT10-mediated viral reactivation. Therefore, the NAT10-expressing plasmid was transduced into iSLK-KSHV cells, and then NAT10 was isolated by immunoprecipitation (IP) for LC–MS/MS analysis. Notably, a lysine lactylation (Kla) site in NAT10 was repeatedly identified (Fig. 2B). IP assay with the pan-lactyl-lysine antibody further confirmed that lactylation occurred on NAT10 in iSLK cells with or without KSHV infection (Fig. 2C). Lactylation modifications appeared on endogenous NAT10 in iSLK-KSHV cells after KSHV reactivation (Fig. 2D, E). After addition of lactic acid to the culture medium of iSLK-KSHV cells, the lactylation level of NAT10 was dramatically elevated (Fig. 2F), which was consistent with the results from the doxycycline-induced iSLK-KSHV cells (Fig. 2G). These results suggest that lactate-driven NAT10 lactylation is enhanced during KSHV reactivation.

**Fig. 3:**
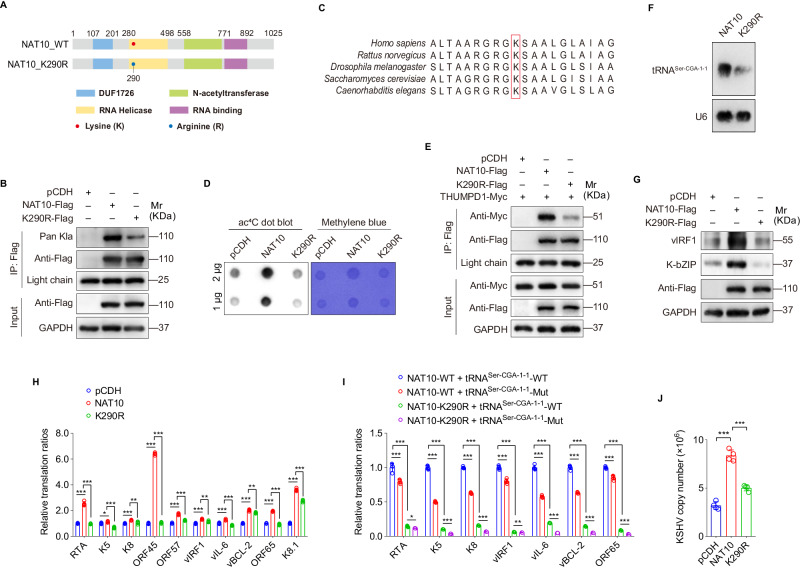
NAT10 lactylation at Lys290 promotes viral mRNAs translation and virion production of KSHV. **A** Schematic representation of DUF1726 domain (marked in blue), RNA helicase domain (marked in yellow), *N*-acetyltransferase domain (marked in green) and RNA-binding domain (marked in purple) of NAT10. The identified lactylation lysine site (**K**) within the RNA helicase domain was marked in red, which was mutated to arginine (**R**) and marked in dark blue. **B** The iSLK-KSHV cells transduced with the wild type (NAT10-Flag), the mutant NAT10 (K290R-Flag) or its control (pCDH) for 48 h were subjected to the anti-Flag immunoprecipitation, and then examined by Western blot with the anti-Pan Kla antibody. **C** The sequences around NAT10 Lys290 from different species were aligned. Conserved Lys290 was marked in red. **D** The total RNAs from cells shown as in (**B**) were subjected to dot blot assay with anti-ac^4^C antibody. Methylene blue staining was used as the internal control. **E** The iSLK-KSHV cells with THUMPD1 overexpression (THUMPD1-Myc) were transduced with the wild type (NAT10-Flag), the mutant NAT10 (K290R-Flag) or its control (pCDH) for 48 h. Cells were subjected to the anti-Flag immunoprecipitation and analyzed by Western blot using anti-Myc antibody. **F** The total RNAs from iSLK-KSHV cells transduced with the wild type (NAT10) and the mutant (K290R) NAT10 for 48 h were subjected to Northern blot with probes to tRNA^Ser-CGA-1-1^, and U6 as the control. **G** The expression levels of vIRF1 and K-bZIP in cells treated as in (**B**) were examined by Western blot with the corresponding antibodies. **H** The RT-qPCR analysis for the ribosome-nascent chain-complex-bound mRNA (RNC-qPCR) of representative viral genes of KSHV (RTA, K5, K8, ORF45, ORF57, vIRF1, vIL-6, vBCL-2, ORF65, and K8.1) in cells shown as in (**B**). *P < 0.05, **P < 0.01 and ***P < 0.001 by Student’s *t*-test. **I** The RT-qPCR analysis for the ribosome-nascent chain-complex-bound mRNA (RNC-qPCR) of representative viral genes of KSHV (RTA, K5, K8, vIRF1, vIL-6, vBCL-2, and ORF65) in iSLK-KSHV cells with the wild type (NAT10-WT) or the mutant (NAT10-K290R) NAT10 overexpression, which were complemented with the wild type (tRNA^Ser-CGA-1-1^-WT) or the mutant (tRNA^Ser-CGA-1-1^-Mut) tRNA^Ser-CGA-1-1^ for 48 h. *P < 0.05, **, *P* < 0.01 and ***P < 0.001 by Student’s *t*-test. **J** By the assessment of ORF26, real-time DNA-PCR for cells treated as in (**B**) was performed to examine viral copy number after doxycycline stimulation for 72 h. ***P < 0.001 by Student’s *t*-test.

Mass spectrometry indicated that Lys290 within the RNA helicase domain of NAT10 was the only lysine residue for its lactylation (Fig. 3A). Then, Lys290 was mutated to arginine to mimic the positive charge state in vivo. Consistently, Lys290 mutation (K290R) decreased the NAT10 lactylation level in iSLK-KSHV cells (Fig. 3B). Since Lys290 is highly conserved among species (Fig. 3C), and the RNA helicase domain is mainly responsible for the RNA acetyltransferase function of NAT10 ^5^, we then examined the role of Lys290-dependent NAT10 lactylation in tRNA ac^4^C modification. Similar to the mutation of Lys290 to alanine in our previous study [14], the results from dot blot indicated that mutation of Lys290 to arginine in the RNA helicase domain weakened the RNA acetyltransferase activity of NAT10 (Fig. 3D), and also blocked the interaction between NAT10 and its adapter protein (Fig. 3E), which is known as THUMP-domain containing protein 1 (THUMPD1) and essential for the acetylation of serine and leucine tRNAs [5]. Meanwhile, Lys290 mutation also decreased the expression level of tRNA^Ser-CGA-1-1^ (Fig. 3F). To further determine the role of NAT10 lactylation on Lys290 in the translation of KSHV lytic transcripts and virus reactivation, we examined the expression of KSHV lytic transcripts and their translation ratios after Lys290 mutation. As expected, the expression of KSHV lytic proteins, such as vIRF1 and K-bZIP, was obviously decreased (Fig. 3G), and the translation ratios of a series of viral lytic transcripts were also decreased (Fig. 3H). All these changes could not be recovered upon non-ac^4^C tRNA^Ser-CGA-1-1^ complement in iSLK-KSHV cells with Lys290 mutation, rather than the ac^4^C-modified tRNA^Ser-CGA-1-1^ (Fig. 3I). Consequently, Lys290 mutation of NAT10 repressed the production of infectious virions (Fig. 3J).

Taken together, these results suggest that NAT10 lysine lactylation at Lys290 promotes the translation of lytic transcripts and virus reactivation via ac^4^C modification on tRNA^Ser-CGA-1-1^.

### ATAT1 functions as a writer of NAT10 lactylation to promote KSHV lytic protein translation and virus reactivation

**Fig. 4:**
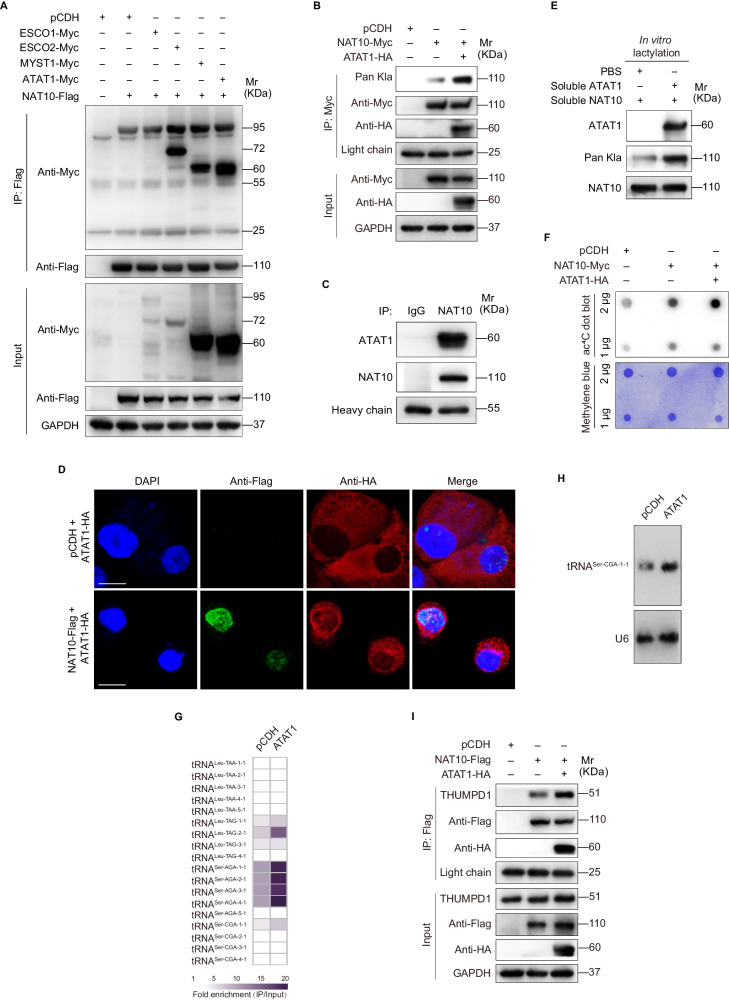
NAT10 is lactylated by ATAT1. **A** The iSLK-KSHV cells transduced with lentiviral NAT10 (NAT10-Flag) or its control (pCDH) were infected by lentiviral ESCO1 (ESCO1-Myc), ESCO2 (ESCO2-Myc), MYST1 (MYST1-Myc), and ATAT1 (ATAT1-Myc) for 48 h, and then subjected to anti-Flag immunoprecipitation. The interaction between NAT10 and acyltransferases was examined by Western blot with anti-Myc antibody. **B** The iSLK-KSHV cells transduced with lentiviral NAT10 (NAT10-Myc) or its control (pCDH) were infected by lentiviral ATAT1 (ATAT1-HA) for 48 h, and then subjected to anti-Myc immunoprecipitation to examine the lactylation level of NAT10 with anti-Pan Kla antibody. **C** Immunoprecipitation assay was performed to examine the interaction between endogenous NAT10 and ATAT1. **D** The iSLK-KSHV cells with ATAT1 (ATAT1-HA) overexpression were infected with lentiviral NAT10 (NAT10-Flag) or its control (pCDH) for 48 h, and then were employed to examine the co-localization of NAT10 and ATAT1 by immunofluorescence staining. Scar bars, 40 μm. **E** ATAT1-catalyzed NAT10 lactylation was determined by mixing soluble ATAT1, NAT10, and lactyl CoA (20 μΜ) in the in vitro lactylation assay. Western blot analysis was performed with the indicated antibodies. **F** The total RNAs from cells treated as in (**B**) were subjected to dot blot assay with anti-ac^4^C antibody. Methylene blue staining was used as the internal control. **G** The heatmap for differentially expressed tRNAs in iSLK-KSHV cells transduced with ATAT1 (ATAT1) or its control (pCDH) for 48 h by tRNA acRIP-seq analysis. The pseudo-color represented as the fold enrichment (IP/Input) of pCDH and ATAT1 group, respectively. Any tRNAs not detected in tRNA acRIP-seq were plotted as white in the heatmap. **H** The total RNAs from cells shown as in (**G**) were subjected to Northern blot with probes to tRNA^Ser-CGA-1-1^ and U6 as the control. **I** The cells treated as in (**B**) were subjected to the anti-Flag immunoprecipitation and analyzed by Western blot using anti-THUMPD1 antibody.

Prior studies have shown that acetyltransferases and deacetyltransferases not only mediate protein acetylation and deacetylation, but also catalyze other acylation modifications, including lactylation [8, 24, 25]. To determine the potential acetyltransferase that is responsible for NAT10 lactylation, co-immunoprecipitation assay (Co-IP) was performed to evaluate the interaction between NAT10 and a series of acetyltransferases, including ESCO1, ESCO2, MYST1, and ATAT1. As shown in Fig. 4A, NAT10 bound to ESCO2, MYST1, and ATAT1, but not ESCO1. However, only ATAT1 overexpression increased the lactylation level of NAT10 (Fig. 4B), while ESCO2 and MYST1 did not show this effect (Fig. S2). Co-IP further confirmed the interaction between endogenous NAT10 and ATAT1 (Fig. 4C). Immunofluorescence staining assay (IFA) also showed the co-localization of NAT10 and ATAT1 in the nuclear area of iSLK-KSHV cells (Fig. 4D). Importantly, in vitro lactylation assay demonstrated that purified ATAT1 lactylated NAT10 in vitro, indicating the activity of ATAT1 as a lactylation writer (Fig. 4E). To determine whether ATAT1-dependent NAT10 lactylation participates in tRNA ac^4^C modification, dot blot was performed. We found that ATAT1 accelerated NAT10-mediated ac^4^C modification in iSLK-KSHV cells (Fig. 4F). ATAT1 overexpression not only increased the ac^4^C modification level of tRNA^Ser-CGA-1-1^ as measured by tRNA acRIP-seq (Fig. 4G) but also augmented tRNA^Ser-CGA-1-1^ expression as measured by Northern blot (Fig. 4H). Moreover, ATAT1 assisted in the interaction between NAT10 and its adapter protein THUMPD1 (Fig. 4I).

**Fig. 5:**
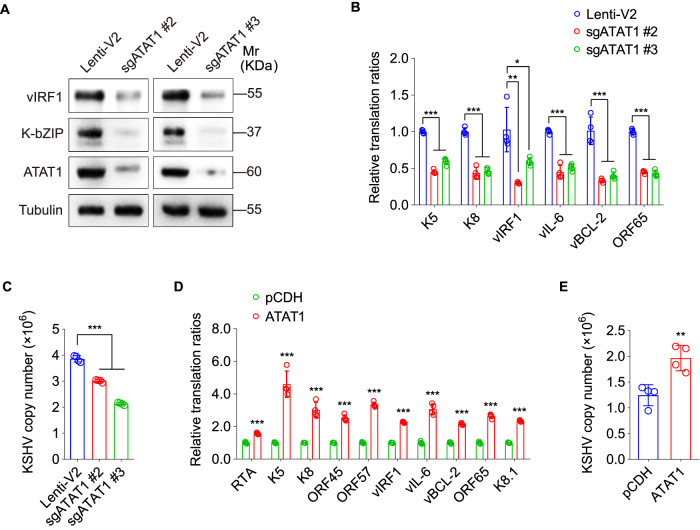
ATAT1, as the lactylation writer of NAT10 promotes viral lytic transcripts translation and virus reactivation. **A** The expression levels of vIRF1 and K-bZIP in iSLK-KSHV cells transduced with lentiviral sgATAT1 (sgATAT1 #2, sgATAT1 #3) or control lentivirus (Lenti-V2) for 72 h were examined by Western blot with the corresponding antibodies. **B** The RT-qPCR analysis for the ribosome-nascent chain-complex-bound mRNA (RNC-qPCR) of representative viral genes of KSHV (K5, K8, vIRF1, vIL-6, vBCL-2, and ORF65) in cells treated as in (**A**). *P < 0.05, **P < 0.01 and ***P < 0.001 by Student’s *t*-test. **C** By the assessment of ORF26, real-time DNA-PCR for cells treated as in (**A**) was performed to examine viral copy number after doxycycline stimulation for 72 h. ***, *P* < 0.001 by Student’s *t*-test. **D** The RT-qPCR analysis for the ribosome-nascent chain-complex-bound mRNA (RNC-qPCR) of representative viral genes of KSHV (RTA, K5, K8, ORF45, ORF57, vIRF1, vIL-6, vBCL-2, ORF65, and K8.1) in iSLK-KSHV cells transduced with lentiviral ATAT1 (ATAT1) or its control (pCDH) for 48 h. ***, *P* < 0.001 by Student’s *t*-test. **E** By the assessment of ORF26, real-time DNA-PCR for cells treated as in (**D**) was performed to detect viral copy number after doxycycline stimulation for 72 h. **P < 0.01 by Student’s *t*-test.

These results indicated that ATAT1 promoted NAT10 lactylation, resulting in tRNA ac^4^C modification; therefore, we speculated that ATAT1-mediated NAT10 lactylation might drive viral lytic transcripts translation and virus reactivation. Although KSHV reactivation had no effect on ATAT1 protein expression (Fig. S3), ATAT1 knockdown repressed the protein levels of KSHV lytic genes and their translation ratios, and eventual the production of infectious virions (Figs. 5A–C, and S4). On the contrary, ATAT1 overexpression boosted KSHV lytic proteins translation and virion production (Figs. 5D, E). Together these data suggest that the acetyltransferase ATAT1 binds to and lactylates NAT10, thus provoking ac^4^C modification on tRNA^Ser-CGA-1-1^ to cause viral lytic transcripts translation and virus reactivation.

### KSHV PAN is responsible for ATAT1 recruitment of NAT10

**Fig. 6:**
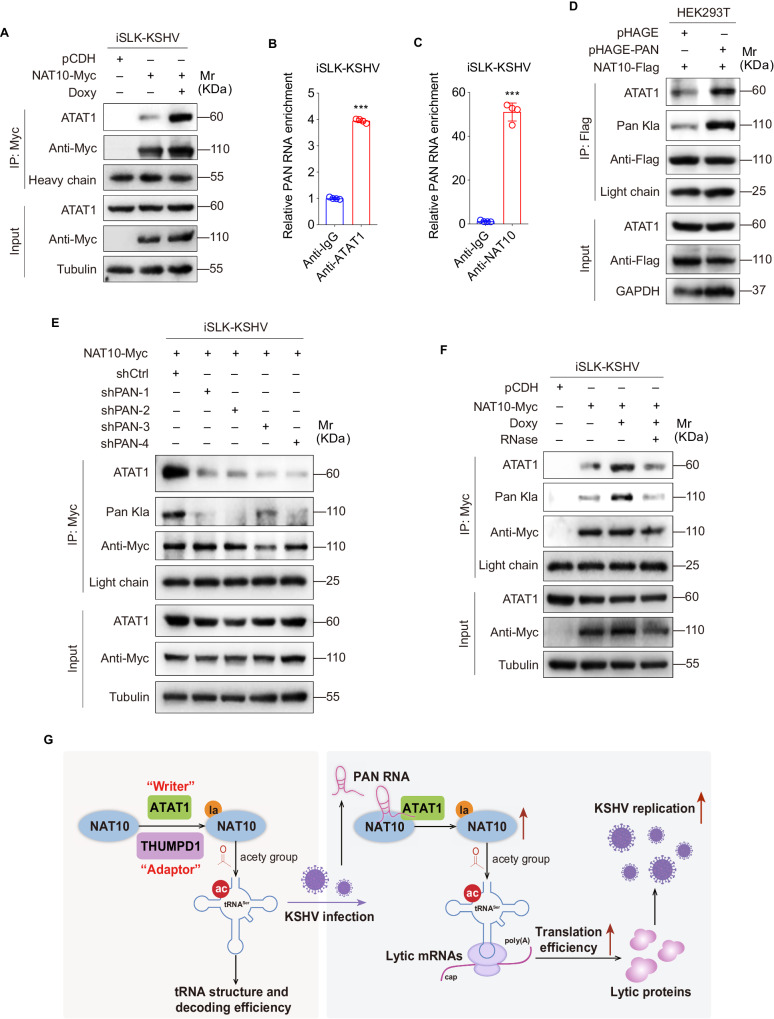
KSHV PAN RNA assists in the recruitment of ATAT1 to NAT10. **A** The iSLK-KSHV cells transduced with lentiviral NAT10 (NAT10-Myc) or control (pCDH) for 48 h were treated with or without doxycycline (Doxy) for 72 h, and then subjected to anti-Myc IP assay and analyzed by Western blot with the anti-ATAT1 antibody. **B**, **C** The iSLK-KSHV cells with doxycycline induction for 72 h were subjected to the anti-ATAT1 (**B**), anti-NAT10 (**C**), or immunoglobulin G (Anti-IgG) RNA immunoprecipitation, and the precipitated PAN RNA were examined by RT-qPCR. ***, *P* < 0.001 by Student’s *t*-test versus the IgG group. **D** HEK293T cells with NAT10 overexpression (NAT10-Flag) were transduced with lentiviral PAN (pHAGE-PAN) or control (pHAGE) for 48 h. Cells were subjected to the anti-Flag immunoprecipitation and analyzed by Western blot using anti-ATAT1 and anti-Pan Kla antibodies. **E** The iSLK-KSHV cells with NAT10 overexpression (NAT10-Myc) were transduced with lentiviral PAN shRNAs (shPAN-1, shPAN-2, shPAN-3, and shPAN-4) or control (shCtrl) for 48 h. Cells were subjected to the anti-Myc immunoprecipitation and analyzed by Western blot using anti-ATAT1 and anti-Pan Kla antibodies. **F** The iSLK-KSHV cells transduced with lentiviral NAT10 (NAT10-Myc) or control (pCDH) for 48 h were treated with or without doxycycline (Doxy) for 72 h, while the induced NAT10-overexpressing cells were further treated with RNase. Cell proteins were subjected to anti-Myc IP assay and detected by Western blot with anti-ATAT1 and anti-Pan Kla antibodies. **G** Schematic illustration for the mechanism of lactylated NAT10-dependent tRNA^Ser-CGA-1-1^ ac^4^C modification during KSHV reactivation. As for NAT10 lactylation, ATAT1 serves as a writer. During KSHV reactivation, PAN RNA assists in the interaction between ATAT1 and NAT10 to promote the lactylation level of NAT10. Lactylated NAT10 occurring on Lys290 mediates tRNA^Ser-CGA-1-1^ ac^4^C modification, and eventually increases the translation efficiency of a series viral lytic transcripts, resulting in progeny virion production and virus reactivation.

To elucidate the mechanism by which KSHV reactivation increases NAT10 lactylation level, Co-IP assay was performed. We found that upon doxycycline treatment, KSHV reactivation enhanced the interaction between NAT10 and ATAT1 (Fig. 6A). PAN RNA is the most abundant transcript of KSHV in the lytic phase [26]. Our previous study has indicated that ac^4^C-modified PAN promotes NAT10 recruitment of IFI16 mRNA to activate inflammasomes [14]. Herein, we hypothesized that PAN might serve for the interaction between NAT10 and ATAT1 during KSHV reactivation. Following RNA immunoprecipitation (RIP)-qPCR, we found that KSHV PAN RNA bound to both endogenous NAT10 and ATAT1 in iSLK-KSHV cells with doxycycline treatment (Fig. 6B, C). Overexpression of PAN RNA in HEK293T cells increased the NAT10-bound ATAT1, and subsequently the lactylation level of NAT10 (Fig. 6D), while silencing PAN RNA exhibited an opposite effect in doxycycline-induced-iSLK-KSHV cells (Figs. 6E and S5). Importantly, after RNase treatment, the PAN RNA-free cell lysis extracted from iSLK-KSHV cells were examined to show decreased bindings between NAT10 and ATAT1, and the subsequently, a low lactylation level of NAT10 (Fig. 6F). These results support the hypothesis that KSHV PAN RNA assists in the interaction between NAT10 and ATAT1, thus enhancing NAT10 lactylation to promote viral lytic protein expression and reactivation.

## Discussion

In epitranscriptomics, post-transcriptional modification of RNA as a critical regulators of gene expression, has slipped into the frontier of research. RNA modifications make up the episodic transcriptome, including *N*^6^-methyladenosine (m^6^A), 7-methylguanosine (m^7^G), 5-methylcytidine (m^5^C) and *N*^4^-acetylcytidine (ac^4^C), etc [1, 27]. Among all RNA modifications, ac^4^C is an ancient and conserved modification mapping to a wide spectrum from archaea bacteria to humans. Both coding and non-coding RNAs are substrates for ac^4^C modification, while tRNA, 18S rRNA and mRNA act as the primary substrates. Yeast tRNA is the first reported substrate for ac^4^C modification [28, 29]. In the 1970s, ac^4^C was observed at the wobble position of tRNA^Met^ in *Escherichia coli*, serving to prevent misreading of codons [30, 31], and ac^4^C was identified in the modified-18S rRNA from rat liver [32, 33]. Subsequently, the dominant sites of ac^4^C in eukaryotes were revealed as helices 34 and 45 of 18S rRNA, which regulate ribosome maturation, and the D-stem of tRNA^Ser^ and tRNA^Leu^, which regulates protein translation efficiency [2–5]. Recently, using antibody-mediated enrichment followed by next-generation sequencing, Arango et al. have proven that the ac^4^C in human mRNAs regulates mRNA stability and translation efficiency [34], which inspires us to reconsider that ac^4^C modification may play different roles in regulating RNA processing events. Our latest study has already identified that ac^4^C modification, occurring in a viral long non-coding RNA as well as a host mRNA, promotes viral reactivation and inflammasome activation during KSHV infection [14]. Here, we further elucidated the role of main RNAs targeted by ac^4^C modification in these processes.

As the first identified and most abundant substrate of ac^4^C modification, tRNAs bridge nucleobase sequence with amino acid sequence during protein synthesis, relying on mechanisms such as transcription, transcript processing, localization and ribonucleotide base modification [35]. Notably, enzyme-catalyzed modifications may occur at specific bases and sugar positions of tRNAs to maneuver the interactions between anticodon and codon, and subsequent translation efficiency and fidelity. To date, over 120 types of tRNA modifications, including ac^4^C modification, have been identified to exert influences on tRNA structural stability and decoding [36, 37]. Following the first reported cytidine acetylation in *E. coli* under the influence of acetyltransferase TmcA [5, 30, 31], conserved ac^4^C marks have been consecutively detected in eukaryotic tRNA^Ser^ and tRNA^Leu^ by high-performance liquid chromatography (HPLC); meanwhile, Kre33 and NAT10 function as essential RNA acetyltransferase enzyme in budding yeast and humans, respectively [5]. Moreover, the interactions of acetyltransferases with adapter proteins, such as Kre33 with Tan1, or NAT10 with THUMPD1, are required for tRNA acetylation [5]. In the previous study, ac^4^C-seq, a chemical genomic method for the transcriptome-wide quantitative mapping of ac^4^C at single-nucleotide resolution, was performed to detect the ac^4^C sites in the three known modified tRNAs, including tRNA^Ser-CGA-1-1^, tRNA^Ser-CGA-4-1^ and tRNA^Leu-TAG-3-1^, while no additional tRNA sites met the detection thresholds for ac^4^C [4], indicating that the ac^4^C modifications in these abundant eukaryotic tRNAs have been well annotated. We utilized the antibody-based tRNA acRIP-seq to further illustrate the abnormal expression of ac^4^C tRNAs upon NAT10 knockdown. Consistently, the enrichment of tRNA^Ser-CGA-1-1^ was significantly decreased, but that of tRNA^Leu-TAG-3-1^ and tRNA^Ser-CGA-4-1^ were not, which may be due to the specificity of cells. Moreover, different from previous studies, our tRNA acRIP-seq also captured other tRNAs that were potentially modified, but showed no difference in abundances after NAT10 knockdown or ATAT1 overexpression. Considering the false-positive results from antibody-based methods and overlooking a rarely possible of NAT10-independent ac^4^C modification, we focused on NAT10-regulated tRNA^Ser-CGA-1-1^ in the further studies, which has been validated through orthogonal methods [4].

To identify the downstream targets of NAT10-mediated tRNAs ac^4^C modification, we detected the ribosome-nascent chaincomplex-bound mRNA, using RT-qPCR, to evaluate the mRNAs that undergo active translation upon modified-tRNAs overexpression. Consequently, only ac^4^C modified-tRNA^Ser-CGA-1-1^, but not tRNA^Leu-TAG-3-1^, increased the translation ratios of KSHV lytic transcripts. Therefore, we further demonstrated the role of tRNA^Ser-CGA-1-1^ in KSHV lytic proteins synthesis, and both in vivo and in vitro assays achieved consistent results from RNC-qPCR. Interestingly, by analyzing the serine ratio of different KSHV lytic proteins, we found that the viral proteins with a higher serine ratio (such as RTA containing 65 serine) were more abundant after challenge with wild-type tRNA^Ser-CGA-1-1^ overexpression, implying that the expression of KSHV lytic proteins relies on tRNA^Ser-CGA-1-1^ with ac^4^C modification.

Mass spectrometry has discovered new patterns of lysine acylations, including lactylation, as well as their role in the pathogenesis and potential in the treatment of infectious diseases [8, 38, 39]. The latest study has found that lactate, a glycolytic product, acts as an epigenetic regulator to stimulate gene transcription via lysine lactylation of histone and non-histone proteins [8, 40, 41]. The energy metabolism in tumor cells features a “Warburg effect” [42], which means even under absolutely aerobic conditions, cancer cells prefer to produce lactate and adenosine triphosphate (ATP), and consume large amounts of glucose via glycolysis rather than oxidative phosphorylation. Increasing evidence has shown that the metabolism in KSHV-infected cells takes on a similar profile to that in cancer cells to support viral replication, cell survival and proliferation [43]. Our previous study has shown that KSHV infection induces aerobic glycolysis, as indicated by increased glucose uptake, ATP and lactate production [22]. Here, we found that lactate was consistently accumulated during KSHV reactivation. Considering that lactate affects histone Kla in a dose-dependent manner [8], we further focus on whether KSHV-induced lactate promotes the lactylation level of NAT10. Consequently, lactate-driven NAT10 lactylation was identified at Lys290, a helicase domain of NAT10 primarily responsible for its RNA acetyltransferase function. We then mutated the Lys290 to arginine to mimic the positive charge state in vivo. The results confirmed lactylation at Lys290 was crucial for the role of NAT10 in tRNAs ac^4^C modification. Nevertheless, the opposite mutation should be performed to mimic the negative charge state in future.

Similar to other post-translational modifications, lactylation is reversible and regulated by acetyltransferases and deacylases. For instance, p300 enzyme catalyzes the transfer of the lactyl group from lactyl-CoA to histones [8]. This study unprecedently showed that ATAT1 serves as a lactylation writer for NAT10. Previously, ATAT1 has been indicated to catalyze acetylation of α-tubulin at lysine 40 in various organisms ranging from *Tetrahymena* to humans [44, 45]. In this study, we showed that acetyltransferase ATAT1 bound to and lactylated NAT10 both in vivo and in vitro. Importantly, KSHV PAN RNA facilitated the interaction between NAT10 and ATAT1. Sequentially, NAT10 was lactylated, ac^4^C modification of tRNA^Ser-CGA-1-1^ was enhanced, and the translation and reactivation of lytic proteins were boosted. Therefore, our findings suggest that ATAT1 may function as a writer for lactylation in addition to acetylation. However, the mechanisms by which lactylation is regulated by ATAT1 remain to be clarified.

PAN, a long non-coding nuclear RNA, accumulates to an exceedingly high level during KSHV reactivation [46]. PAN RNA is rarely expressed in the latent phase [47, 48], but its expression level increased by more than 1,000-fold in the lytic phase [49]. Importantly, PAN is required for the expression of a subset of KSHV lytic genes as well as the production of infectious virions [50]. Our previous study has demonstrated that NAT10 arouses its ac^4^C modification by targeting PAN [14]. Interestingly, the current study indicated that PAN mediated the interaction between NAT10 and ATAT1 to enhance the lactylation of NAT10, implying a complex network of RNAs post-transcriptional modifications and proteins post-translational modifications, as well as a new mechanism governing PAN-dependent KSHV gene expression in the lytic phase. However, a KSHV virus mutated by deleting PAN is still needed to further confirm its potential effect on NAT10 lactylation.

Taken together, our study unprecedently identifies a lysine lactylation occurring on NAT10 and catalyzed by acetyltransferase ATAT1 as a writer. Lactylated NAT10 exhibits its RNA acetyltransferase function to facilitate ac^4^C modification and enhance tRNA^Ser-CGA-1-1^ expression. During KSHV infection, the most abundant viral transcript PAN RNA mediates the interaction of NAT10 and ATAT1 to promote NAT10 lactylation and tRNA^Ser-CGA-1-1^ ac^4^C modification, ultimately increasing the efficiency of KSHV lytic protein translation and virus reactivation (Fig. 6G). These findings highlight the mechanisms by which tRNA ac^4^C modification cooperates with lysine lactylation during DNA virus infection, and provide the potential therapeutic implications for KSHV infection and related malignancies.

## Materials and methods

### Cell culture and reagents

NAT10^+/-^ iSLK-KSHV cells obtained and validated in our previous study were cultured in high-glucose DMEM with 10% fetal bovine serum (FBS), 1 μg/mL puromycin (Beyotime, China), 250 μg/mL G418 (Biofroxx, China), and 1.2 mg/mL hygromycin B (BBI, China) [14]. As the KSHV-negative control of iSLK-KSHV cells, iSLK-Puro cells were grown under the same conditions but without hygromycin B supplement. Above mentioned cell lines were kindly provided by Dr. Ke Lan from State Key Laboratory of Virology, Wuhan University (Wuhan, China). HEK293T were purchased from ATCC (CRL-11268) and transfected with the Lipofectamine 2000 Reagent (Invitrogen, USA). All cells were cultured in a 5% CO_2_ incubator at 37 °C and negative for mycoplasma contamination by Myco-Blue Mycoplasma Detector (D103-01/02, Vazyme Biotech Co., Ltd, Nanjing, China). RNase was purchased from Beyotime Biotechnology (ST576, China), and L-(+)-Lactic acid was from Sigma (L6402, USA).

### Plasmids construction

To construct tRNA overexpression plasmids, the U6 promoter was firstly removed from the pLKO.1 lentiviral expression vector, while a SalI RE site was then introduced into the multiple cloning site. The genomic sequence spanning the individual tRNA, including 350 nt upstream and 150 nt downstream sequence (containing the Pol III promoter, upstream leader, tRNA, and downstream trailer sequences) was subcloned and inserted into MluI and SalI RE sites in the flanking regions of the amplicon. Based on the tRNA overexpression plasmids, the tRNA mutant plasmids were generated by substituting C in position 12 of tRNA^Ser-CGA-1-1^ and tRNA^Leu-TAG-3-1^ to T, and constructed by GenScript Biotech Corporation (Nanjing, China). The NAT10-Myc, ESCO1-Myc, ESCO2-Myc, MYST1-Myc and ATAT1-Myc were generated based on the pCDH vector (Tsingke Biotechnology Co., Ltd., China). The NAT10 overexpression plasmid (pCDH-Flag-NAT10) was constructed as previously described [14], while the lysine at position 290 of the amino acids encoded by NAT10 were mutated to arginine by Tsingke Biotechnology Co., Ltd. (Beijing, China). The short hairpin RNA (shRNA) expressing plasmids of PAN RNA were generated based on the mpCDH plasmid, and the sequences were listed in Table S1. All plasmids were prepared using Vazyme FastPure Plasmid Mini Kit and confirmed by DNA sequencing.

### CRISPR-Cas9 system

CRISPR-Cas9 system was utilized to construct the knockdown of ATAT1 in iSLK-KSHV cells. The guidance RNAs were designed according to the online software (chopchop.cbu.uib.no), and the sequences were listed in Table S1. A single guide RNA (sgRNA) was annealed and cloned into lentiCrispr V2 vector (Lenti-V2). HEK293T cells were co-transfected with guide RNA plasmids, the packaging plasmid (psPAX2) and the envelope plasmid (pMD2.G) for 48 h, and viral supernatants were collected. After infection, cells were screened with puromycin (1 μg/mL).

### tRNA ac^4^C-seq

tRNA ac^4^C-seq was mainly provided by Cloud-Seq Biotech (Shanghai, China). Briefly, the RNA immunoprecipitation (RIP) assay was carried out with GenSeq^®^ ac^4^C RIP kit (GenSeq Inc., China) according to the manufacturer’s instructions. The remaining RNA was size-selected for <200 nt RNA fraction with the MirVana Isolation Kit (Invitrogen). The RNAs were then de-aminoacylated in 0.1 M Tris-HCl pH 9.0 and 1 mM EDTA for 30 min at 37 °C. tRNA libraries were constructed according to TruSeq Small RNA Preparation Kit (Illumina) instructions. All libraries were size-selected (170-210 bp) before sequencing and then sequenced in a HiSeq platform (Illumina).

### Illumina sequencing & bioinformatic data

The tRNA library was firstly adapted from the tRNAScan-SE library by appending CCA to tRNAs from the genomic tRNA database (http://gtrnadb.ucsc.edu/GtRNAdb2/genomes/). Isodecoders with identical scores were consolidated for ease of identity assignment, decreasing the number of reference genes and pseudogenes. Then, raw reads were generated after sequencing, image analysis, base calling and quality filtering on Illumina sequencer. Q30 was used to perform quality control. The adapter sequences were trimmed and the adapter-trimmed-reads (at least 15 nt) were left by cutadapt software (v1.9.3). Then, trimmed reads from all samples were aligned using bowtie2 software to the aforementioned tRNA library with sensitive options. Mapped reads on each tRNA were counted using samtools. IP and Input raw expression data were normalized by TPM method, then IP/IgG was calculated as tRNA expression level base on normalized results. *t*-test was used to detect statistical differences in the expression level of tRNA.

### Northern blot

Total RNA (10 μg) was added with an equal volume of RNA Loading Buffer (Takara, #9168) and heated at 65 °C for 10 min. Then the denatured RNA was electrophoresis on 2% Tris-borate-EDTA (TBE) agarose gel in cold 1 × MOPS buffer at 60 V for 2 h. The RNA was then transferred to nylon membrane at 60 V in 0.5 × TBE with 20% methanol for 1 h at 4 °C. The RNA was crosslinked to the membrane by UV irradiation (120 mJ/cm^2^), and then the membranes were incubated with biotin-labeled probes overnight at 42 °C and rinsed with hybridization washing buffer for 30 min at 42 °C. After blocking, the membranes were incubated with Streptavidin-HRP conjugate and signals developed using a chemiluminescence imaging system. The sequences of probes were listed in Table S1.

### Ribosome-nascent chain complex-qPCR (RNC-qPCR)

The RNC extraction was performed as previous described [51]. Briefly, cells were pre-treated with 100 μg/mL cycloheximide for 15 min, followed by pre-chilled phosphate buffered saline (PBS) washing and addition of 1 mL cell lysis buffer [1% Triton X-100 in ribosome buffer (RB buffer) [20 mM HEPES-KOH (pH 7.4), 15 mM MgCl_2_, 200 mM KCl, 100 μg/mL cycloheximide and 2 mM dithiothreitol]. After bathing on ice for 30 min, cell lysates were scraped and transferred to pre-chilled 1.5 mL tubes. Cell debris was removed by centrifuging at 16, 200 × *g* for 10 min at 4 °C, and the supernatants were transferred from the upper layer of 35 mL sucrose buffer (30% sucrose in RB buffer). After ultra-centrifugation at 174, 900 × *g* for 5 h at 4 °C in a SW32 rotor (Beckman Coulter, USA), the RNC pellets that contain the polysome fractions were collected, and then the RNA samples were isolated from the input and RNC samples for RT-qPCR.

### Virion release assay

1 μg/mL doxycycline was added into the indicated iSLK-KSHV cells for 72 h to stimulate KSHV lytic reactivation. The supernatant was collected and added with 1 μL DNase I for digesting linear DNA molecules by 37 °C water bathing for 1 h. The progeny virus DNA in the supernatant was extracted with a micro-sample genomic DNA extraction kit, and determined by absolute quantification, while the standard curve Ct value versus genome copy number was generated with detection of KSHV ORF26.

### RNA extraction and RT-qPCR analysis

Total RNA was extracted using TRIzol reagent (Invitrogen) according to the standard procedure, and then reverse transcribed by HiScript III RT SuperMix (Vazyme Biotech Co., Ltd). The mRNA was analyzed by RT-qPCR with ChamQ SYBR qPCR Master Mix (Vazyme Biotech Co., Ltd) on StepOnePlus™ Real-Time PCR System (Applied Biosystems), and the sequences of specific primers were listed in Table S1. The results were analyzed using the ^ΔΔ^Ct method and normalized to GAPDH.

### LC–MS/MS

LC–MS/MS was mainly provided by Beijing Bio-Tech Pack Technology Company Ltd. Briefly, samples were analyzed on a Q Exactive^TM^ Hybrid Quadrupole-Orbitrap^TM^ Mass Spectrometer (Thermo Fisher Scientific) coupled with a high-performance liquid chromatograph (EASY-nLC 1200 system, Thermo Fisher scientific). Dried peptide samples were dissolved in solvent A (0.1% formic acid in water) and loaded onto a trap column (150 μm i.d. × 5 cm, packing: Reprosil-Pur 120 C18-AQ 3 μm) with a maximum pressure of 300 bar using solvent A, then separated on a home-made 100 μm × 18 cm silica microcolumn (packing: Reprosil-Pur 120 C18-AQ 3 μm) with a gradient of 4–95% mobile phase B (0.1%FA, 80% ACN) at a flow rate of 600 nl/min for 66 min. Ms analysis was conducted with one full scan (300-1 800 *m*/*z*, *R* = 70,000 at 200 *m*/*z*) at an automatic gain control target of 3e6 ions, followed by up to 20 data-dependent Ms/Ms scans with higher energy collision dissociation target 1e5 ions, max injection time 50 ms, isolation window 3 mz, normalized collision energy of 28%. The detector was done using Orbitrap (R = 17,500 at 200 *m*/*z*). Data were acquired using the Xcalilbur software (Thermo Fischer Scientific).

### Western blot and antibodies

Western blot was performed as previous described [52, 53]. Anti-NAT10 (ab194297) and anti-ac^4^C (ab252215) were purchased from Abcam. Anti-Myc mouse antibody, anti-HA mouse antibody and anti-Flag mouse antibody were purchased from MEDICAL & BIOLOGICAL LABORATORIES CO., LTD (Japan). Anti-l-Lactyl lysine rabbit antibody (PTM-1401RM) was obtained from PTM Biolabs (Hangzhou, Zhejiang, China). Anti-GAPDH (sc-47724), anti-Tubulin (sc-23948) and anti-K-bZIP (sc-69797) were from Santa Cruz Biotechnology. Anti-ATAT1 (orb688509) and anti-THUMPD1 (14921-1-AP) were from Biorbyt and Proteintech, respectively. The polyclonal rabbit anti-vIRF1 antibody was from Dr. Gary Hayward from Viral Oncology Program, The Johns Hopkins School of Medicine (Baltimore, Maryland, USA) [54–57].

### RNA immunoprecipitation (RIP)

RIP assay was performed using Magna RIP Kit (17-701, Millipore) according to the manufacturer’s instructions. Briefly, 5 μg anti-ATAT1 (orb688509), anti-NAT10 (ab194297) or anti-rabbit IgG (Millipore, Germany) were incubated with 50 μL magnetic beads and then added into cell lysates (~10^7^ cells per sample). The RNA-protein IP complexes were washed for 6 times and incubated with proteinase K to isolate immunoprecipitated protein-bound RNA. Finally, the released RNA was purified by phenol-chloroform RNA extraction for qPCR analysis. Normalization of the relative enrichment was done to the input as: %Input = 2^-(Ct [IP] – (Ct [input]-LOG2[10]). Amplification primers for PAN RNA were listed in Table S1.

### In vitro translation

PCR was performed to generate DNA fragments with T7 promoter, Kozak sequence, and CDS region of the detected genes, and the sequences of specific primers were listed in Table S1. tRNA overexpressing plasmids of the wild type and mutant tRNA^Ser-CGA-1-1^ were transfected into HEK293T cells and purified by DNA probe digestion. Biotinylated DNA oligonucleotide probes were synthesized for the target tRNA, and the hybrid tRNA oligonucleotide complexes were enriched using streptavidin magnetic beads. After adding TRIzol reagent, RNA was extracted and digested using DNase I to release the target tRNA. Finally, the purified DNA template and tRNA were used for in vitro translation experiment with using the PURExpress Δ (aa, tRNA) Kit (NEB #E6840) according to the manufacturer’s instructions. Then, protein loading buffer was added to the system and boiled for 5 min. Western blot were performed to detect the translation efficiency of indicated proteins.

### In vitro lactylation

Recombinant NAT10 protein (2 μg, active motif #81376), ATAT1 protein (0.5 μg, # 50072, BPS bioscience) and lactyl CoA (20 μΜ) were incubated in reaction buffer (50 mM HEPES at pH 7.8, 30 mM KCl, 0.25 mM EDTA, 5.0 mM MgCl_2_, 5 mM sodium butyrate, 2.5 mM DTT) at 30 °C for 30 min. Subsequently, SDS loading buffer was added to terminate the reaction before Western blot.

### Statistical analysis

GraphPad Prism 8 was used to calculate the standard deviation of the data. Unpaired two-tailed *t*-test was used for two groups comparisons. *P* < 0.05 was considered statistically significant. All the experiments were repeated no less than three biological times. No statistical methods were used to predetermine sample size. Sample sizes for relevant experiments were determined by power analyses conducted during experiment planning. Data shown represent mean ± SD determined from one representative experiment unless otherwise stated.

## Supplementary information

Supplemental Figure Legends Information

Supplemental Figure S1

Supplemental Figure S2

Supplemental Figure S3

Supplemental Figure S4

Supplemental Figure S5

Supplemental Table S1

Original data of WB, NB and Dot blot

## Data Availability

The tRNA acRIP-seq data generated in this study have been deposited in the NCBI Gene Expression Omnibus (GEO) database under accession number GSE248740. The LC-MS/MS data have been deposited to the ProteomeXchange Consortium (http://proteomecentral.proteomexchange.org) via the iProX partner repository with the dataset identifier PXD049447. Uncropped Western blots and Northern blots are provided as the Supplementary Material.
