# Supplementary material for: Lactylation of NAT10 promotes N4‐acetylcytidine modification on tRNASer-CGA-1-1 to boost oncogenic DNA virus KSHV reactivation
Source: Cell Death Differ. 2024 Jun 15;31(10):1362–74. doi: 10.1038/s41418-024-01327-0 (PMC11445560; doi:10.1038/s41418-024-01327-0)
Supplement: Supplementary file 1 — Supplemental Figure Legends Information [file 41418_2024_1327_MOESM1_ESM.docx]

## Supplemental Figure Legends Information

**Figure S1. Effect of tRNA^Leu-TAG-3-1^ ac^4^C modification on KSHV lytic transcripts translation.**

The RT-qPCR analysis for the ribosome-nascent chain-complex-bound mRNA (RNC-qPCR) of representative viral genes of KSHV (**K5**, **K8**, **vIRF1**, **vIL-6**, **vBCL-2**, and **ORF65**) in iSLK-KSHV cells with overexpression of the wild type tRNA^Leu-TAG-3-1^ (**tRNA^Leu-TAG-3-1^-WT**), mutant tRNA^Leu-TAG-3-1^ (**tRNA^Leu-TAG-3-1^-Mut**), or their control empty vector (**tRNA-EV**) for 48 h. *n.s*, not significant.

**Figure S2. Effects of ESCO2 and MYST1 on NAT10 lactylation.**

The iSLK-KSHV cells transduced with lentiviral NAT10 (**NAT10-Flag**) or its control (**pCDH**) were infected with lentiviral ESCO2 (**ESCO2-Myc**; **A**) or MYST1 (**MYST1-Myc**; **B**) for 48 h, and then subjected to anti-Flag immunoprecipitation to examine the lactylation level of NAT10 using anti-Pan-lactyl-lysine antibody (**Pan Kla**).

**Figure S3. Effect of KSHV reactivation on ATAT1 protein level.**

The protein expression level of ATAT1 in iSLK-KSHV cells during different KSHV reactivation time was examined by Western blot.

**Figure S4. Verification of ATAT1 knockdown efficiency.**

The iSLK-KSHV cells were transduced with lentiviral sgATAT1 (**#1**, **#2**, and **#3**) or control (**Lenti-V2**) for 72 h, and the protein levels of ATAT1 were examined by Western blot.

**Figure S5. Verification of PAN RNA knockdown efficiency.**

The expression levels of PAN RNA in iSLK-KSHV cells transduced with lentivirus-mediated short hairpin RNAs (shRNA) targeting PAN RNA (**shPAN-1~4**) or its control (**mpCDH**) for 48 h were examined by RT-qPCR. ***, *P* < 0.001 by Student’s *t*-test.
