# Supplementary material for: Lactylation of NAT10 promotes N4‐acetylcytidine modification on tRNASer-CGA-1-1 to boost oncogenic DNA virus KSHV reactivation
Source: Cell Death Differ. 2024 Jun 15;31(10):1362–74. doi: 10.1038/s41418-024-01327-0 (PMC11445560; doi:10.1038/s41418-024-01327-0)
Supplement: Supplementary file 7 — Supplemental Table S1 [file 41418_2024_1327_MOESM7_ESM.docx]

**Table S1. Oligonucleotides used for RT-qPCR, PCR, CRISPR/Cas9, shRNAs and Northern blot**

| **RT-qPCR primers** | |
| --- | --- |
| GCGCAAGATGACAAGGGTAAG | KSHV RTA-F |
| CGAGAGGCCGACGAAGC | KSHV RTA-R |
| GCTCCATCTCCGGCCACA | KSHV K5-F |
| ACTTGGCTAACAGTGTCTCGAA | KSHV K5-R |
| TGACGAACATCTGCCATGCTCA | KSHV K8-F |
| TAGTCGCCTCTTGGATCTGC | KSHV K8-R |
| CTCCTCGTCTGTGTCACTCCC | KSHV ORF45-F |
| ACCAAACTTAAGCCGCAAAGCA | KSHV ORF45-R |
| TGGCGAGGTCAAGCTTAACTTC | KSHV ORF57-F |
| CCCCTGGCCTGTAGTATTCCA | KSHV ORF57-R |
| GTCTCTGCGCCATTCAAAAC | KSHV vIRF1 (K9)-F |
| CCGGACACGACAACTAAGAA | KSHV vIRF1 (K9)-R |
| GTATTCTAGAGCCCGCTGCTA | KSHV vIL-6 (K2)-F |
| TTAAATCCTATTAACCCGCAG | KSHV vIL-6 (K2)-R |
| ATTCATGGCCTGTGGATTAAACGA | KSHV vBCL-2-F |
| CTTGTCTCGCATTAAGCCTGT | KSHV vBCL-2-R |
| ATATGTCGCAGGCCGAATAC | KSHV ORF65-F |
| CCACCCATCCTCCTCAGATA | KSHV ORF65-R |
| AAAGCGTCCAGGCCACCACAGA | KSHV K8.1-F |
| GGCAGAAAATGGCACACGGTTAC | KSHV K8.1-R |
| AGCCGAAAGGATTCCACCAT | KSHV ORF26-F |
| GCTGCGGCACGACCAT | KSHV ORF26-R |
| GAAGGTGAAGGTCGGAGTC | GAPDH-F |
| GAAGATGGTGATGGGATTTCC | GAPDH-R |
| GCCGCTTCTGGTTTTCATTG | KSHV PAN-F (for RIP) |
| TTGCCAAAAGCGACGCA | KSHV PAN-R (for RIP) |
|  | |
| **PCR primers** | |
| GCGAATTAATACGACTCACTATAGGGCTTAAGTATAAGGAGGAAAAAATatggcgcaagatgacaagggtaagaag | KSHV RTA-F (for *In vitro* translation) |
| AAACCCCTCCGTTTAGAGAGGGGTTATGCTAGTTACTTATCGTCGTCATCCTTGTAATCgtctcggaagtaattacgccattg | KSHV RTA-R (for *In vitro* translation) |
| GCGAATTAATACGACTCACTATAGGGCTTAAGTATAAGGAGGAAAAAATatggacccaggccaaagaccgaaccct | KSHV vIRF1-F (for *In vitro* translation) |
| AAACCCCTCCGTTTAGAGAGGGGTTATGCTAGTTACTTATCGTCGTCATCCTTGTAATCttgcatggcatcccataacggctt | KSHV vIRF1-R (for *In vitro* translation) |
| GCGAATTAATACGACTCACTATAGGGCTTAAGTATAAGGAGGAAAAAATatgtccaactttaaggtgagagacccc | KSHV ORF65-F (for *In vitro* translation) |
| AAACCCCTCCGTTTAGAGAGGGGTTATGCTAGTTACTTATCGTCGTCATCCTTGTAATCtttctttttgccagaggggggttt | KSHV ORF65-R (for *In vitro* translation) |
| AGAATCGCTTGAAACCAGGA | Validation of CRISPR editing-F |
| TTCCCAGGACTGACTGCCTA | Validation of CRISPR editing-R |
|  | |
| **Probes for Northern blot** | |
| GGATTCGAACCTGCGCGGGGAGAC | tRNA^Ser-CGA-1-1^ |
| GGATTCGAACCTGTGCGGGGAAAC | tRNA^Ser-CGA-4-1^ |
| GAGCCTAAATCCAGCGCCTTAGACCAC | tRNA^Leu-TAG-3-1^ |
| GTAGTCGGCAGGATTCGAACCTG | tRNA^Ser-AGA^ |
| CCAGGAGTGGGGTTCGAACCC | tRNA^Leu-TAA^ |
| AGTATATGTGCTGCCGAAGCGAGCACT | U6 |
|  | |
| **shRNA of KSHV PAN** | |
| TTGCGGGTTATTGCATTGGAT | shPAN-1 |
| ACGTGTCTGAATGTGGAATAT | shPAN-2 |
| GAGGTGGCATTTGTCAGAAGT | shPAN-3 |
| TTGGAAGTTCCAGGCTAATAT | shPAN-4 |
|  | |
| **CRISPR guide RNAs for cloning into LentiCRISPRv2** | |
| GCGAGTGGAACCGCACCAAC | ATAT1-1 |
| ATGACCATTATAGATGAACT | ATAT1-2 |
| TGATGCACTAGTGATAGGAG | ATAT1-3 |

F, Forward; R, Reverse
