# Supplementary material for: Lactylation of NAT10 promotes N4‐acetylcytidine modification on tRNASer-CGA-1-1 to boost oncogenic DNA virus KSHV reactivation
Source: Cell Death Differ. 2024 Jun 15;31(10):1362–74. doi: 10.1038/s41418-024-01327-0 (PMC11445560; doi:10.1038/s41418-024-01327-0)

Figure 1A

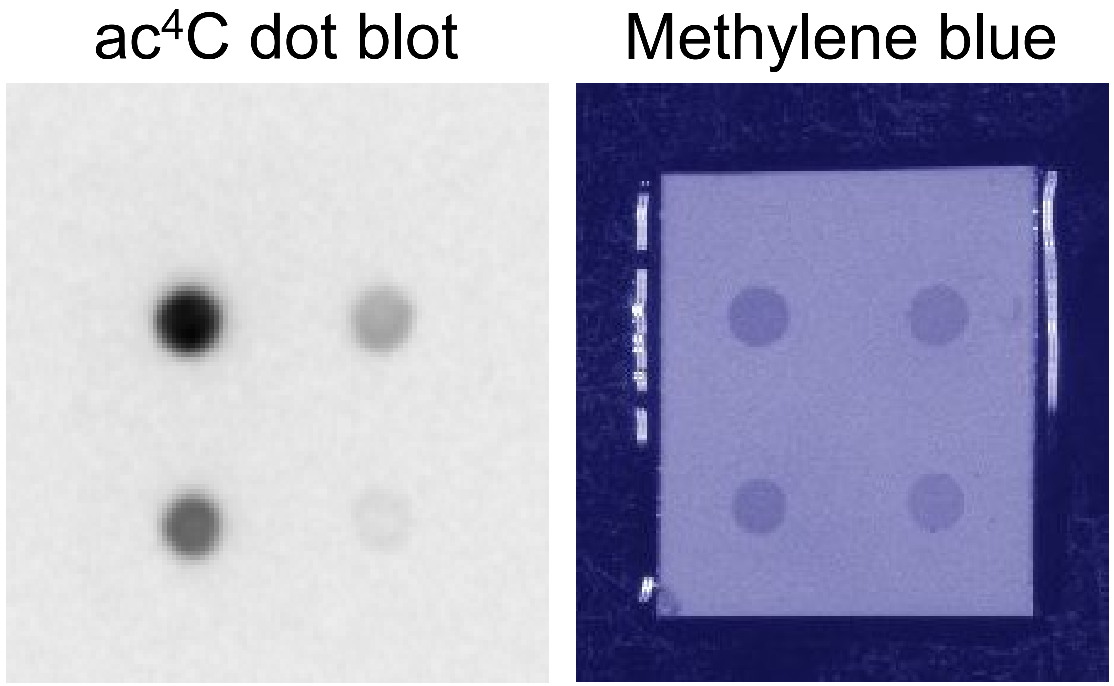

Figure 1C

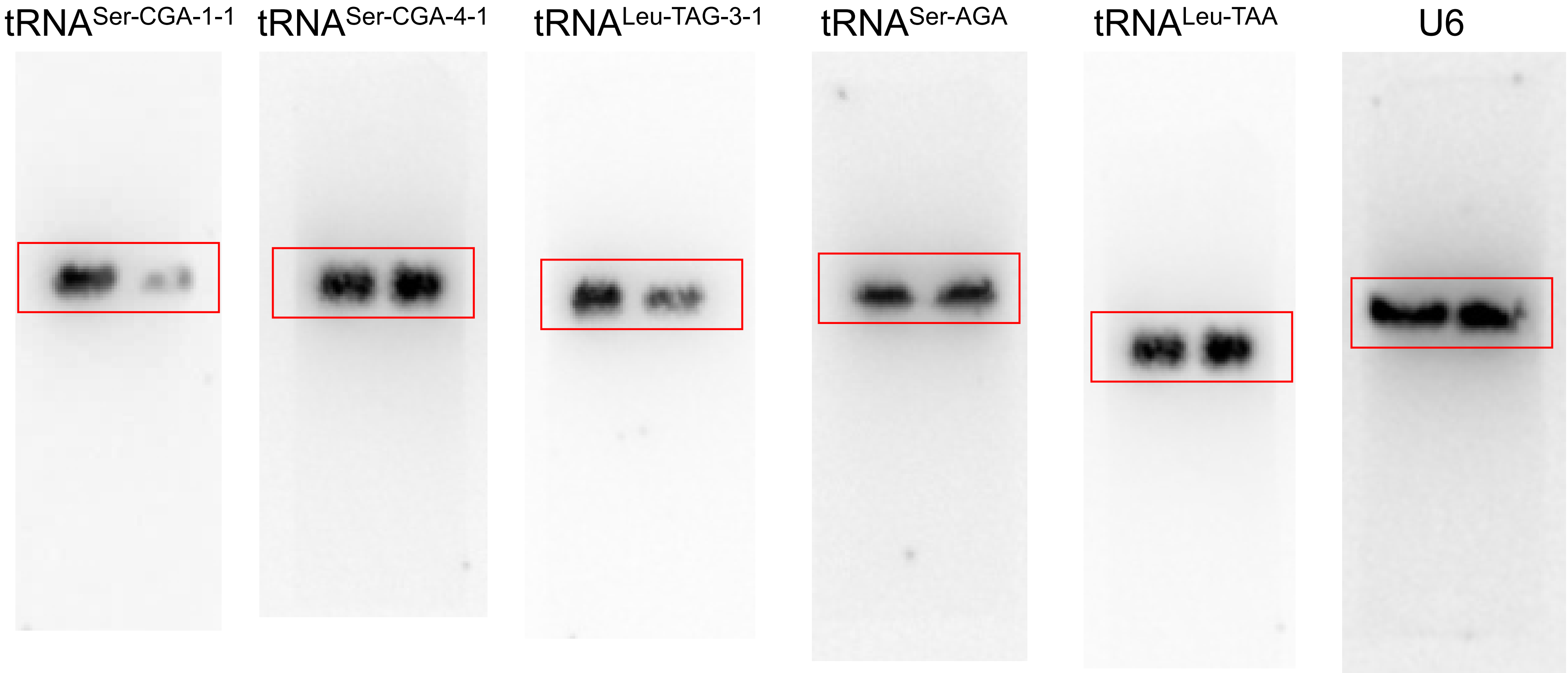

tRNA^Ser-CGA-1-1
tRNA^Ser-CGA-4-1
tRNA^Leu-TAG-3-1
tRNA^Ser-AGA
tRNA^Leu-TAA
U6

Figure 1F

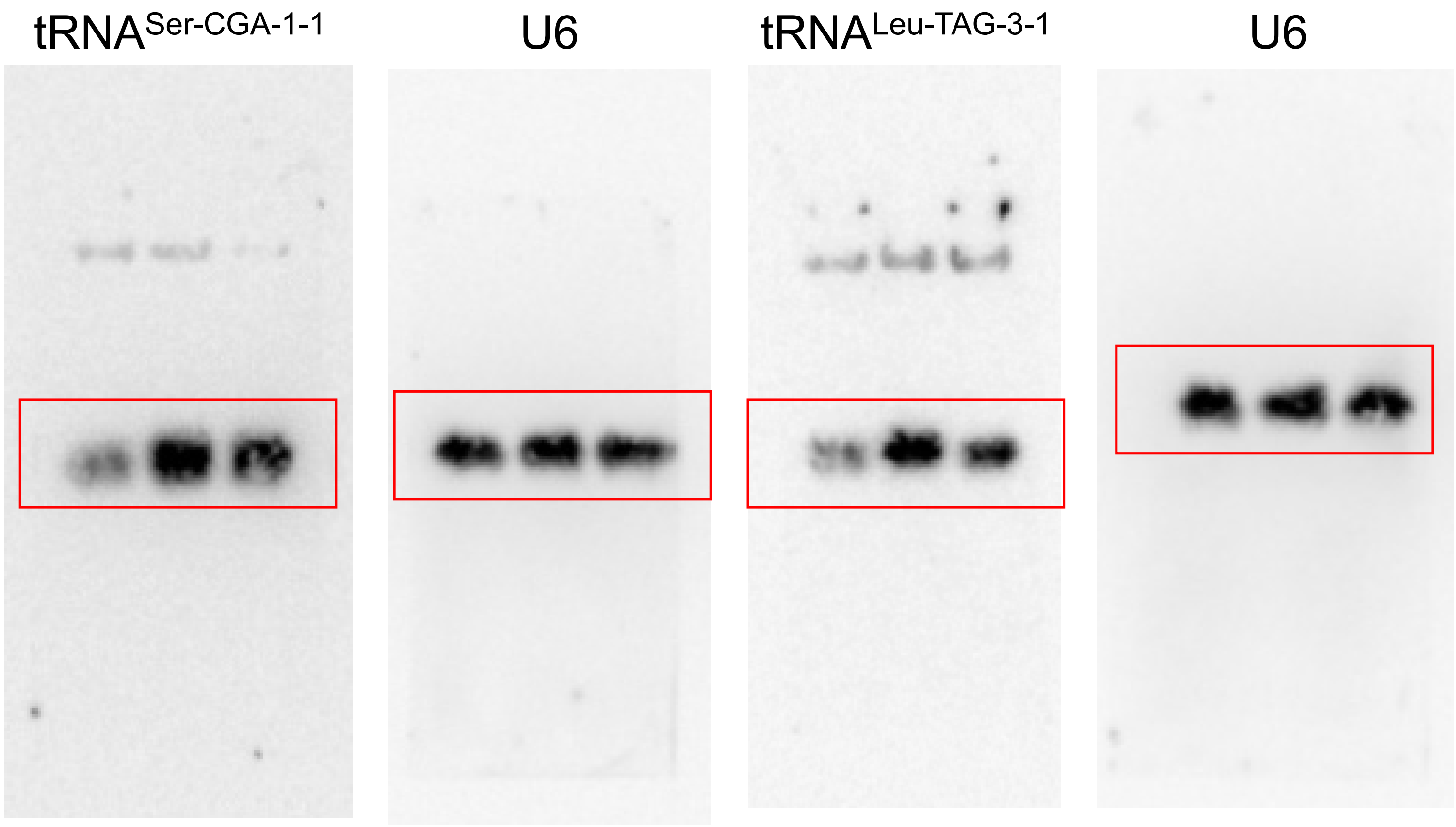

Figure 1H

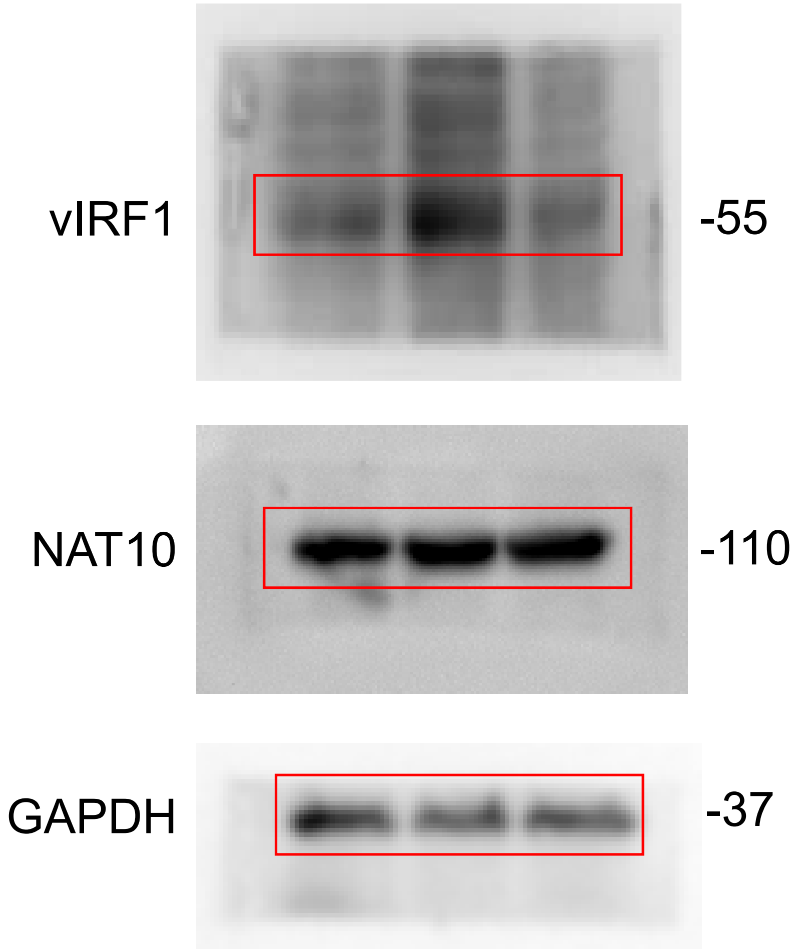

Figure 1I

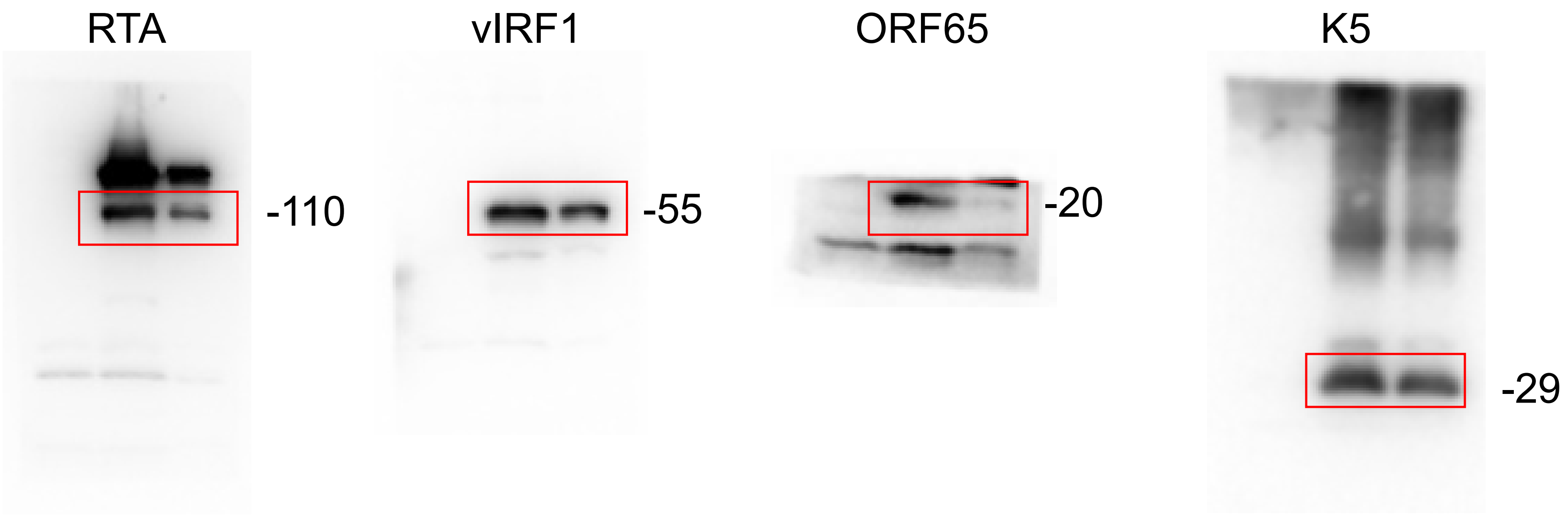

Figure 2C

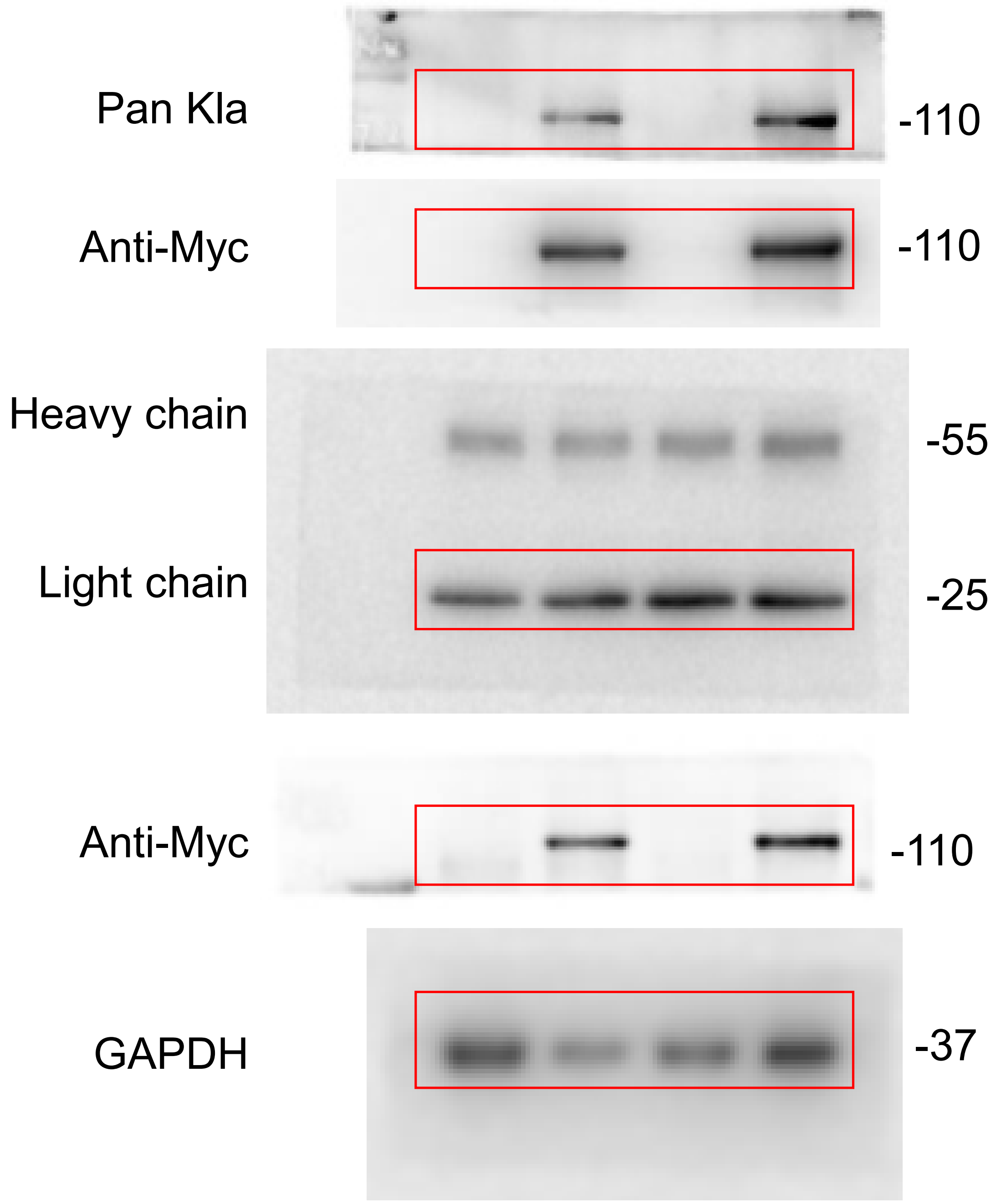

Pan Kla
-110
Anti-Myc
-110
Heavy chain
-55
Light chain
-25
Anti-Myc
-110
GAPDH
-37

Figure 2D

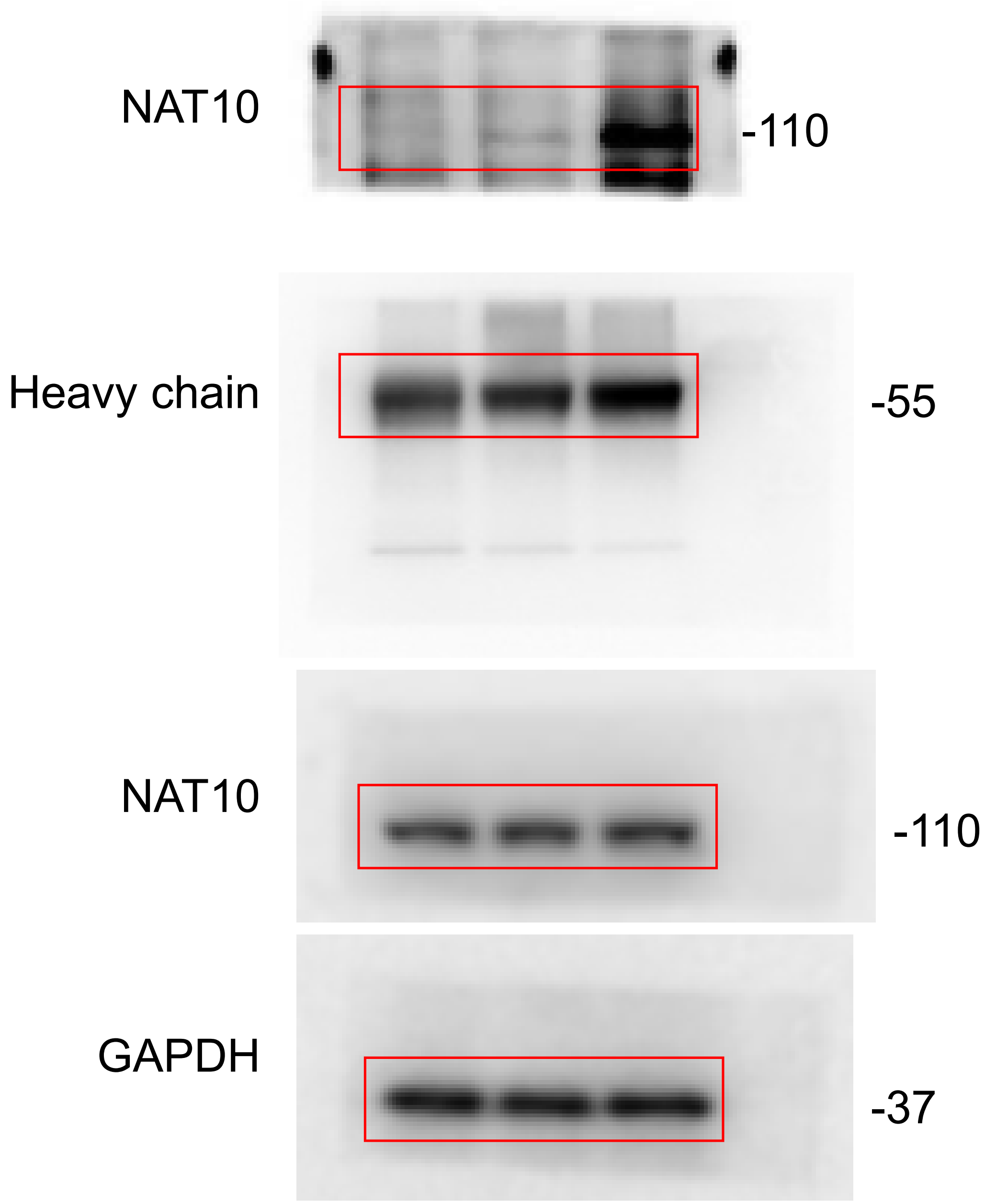

Figure 2E

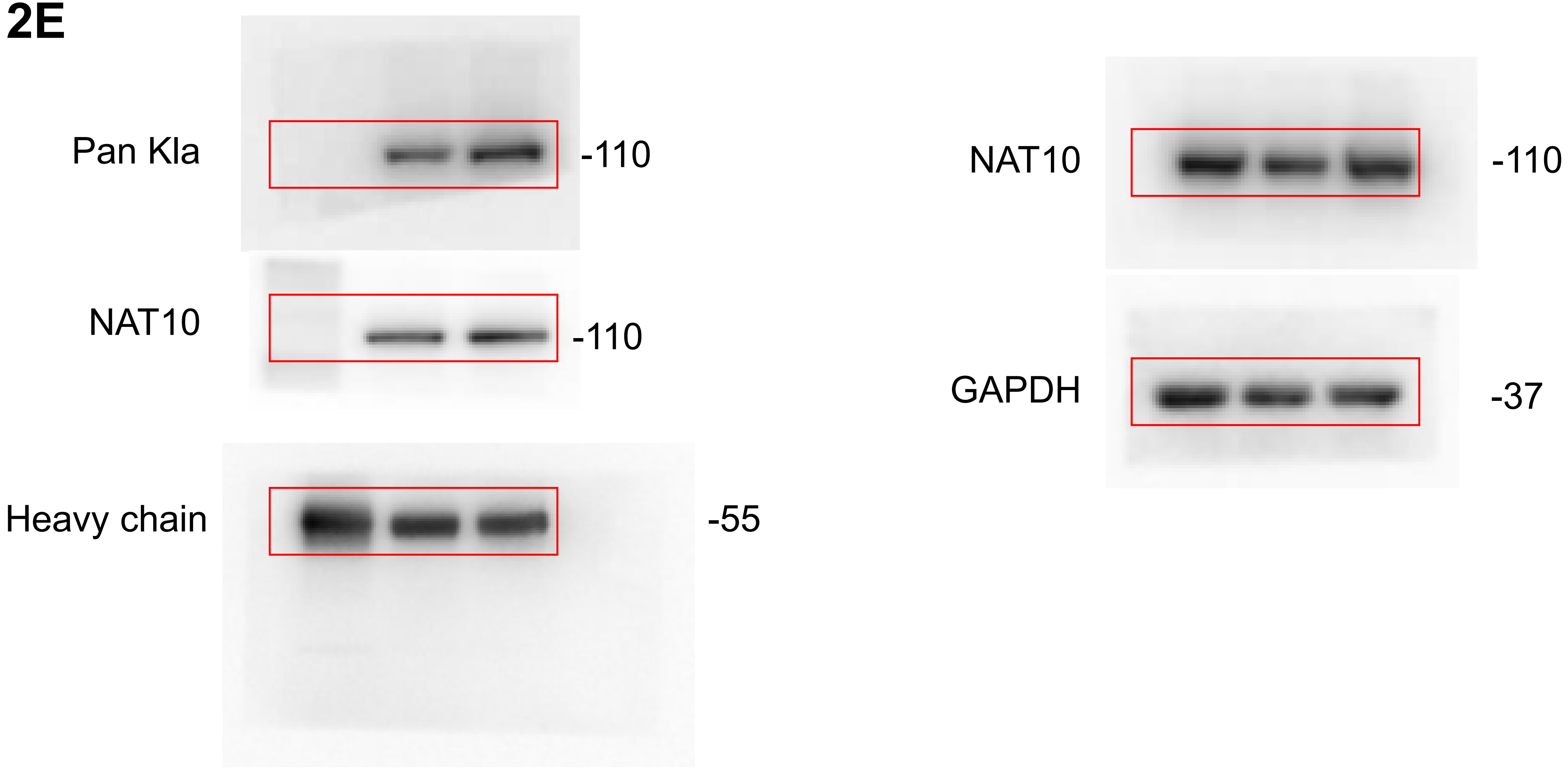

Figure 2F

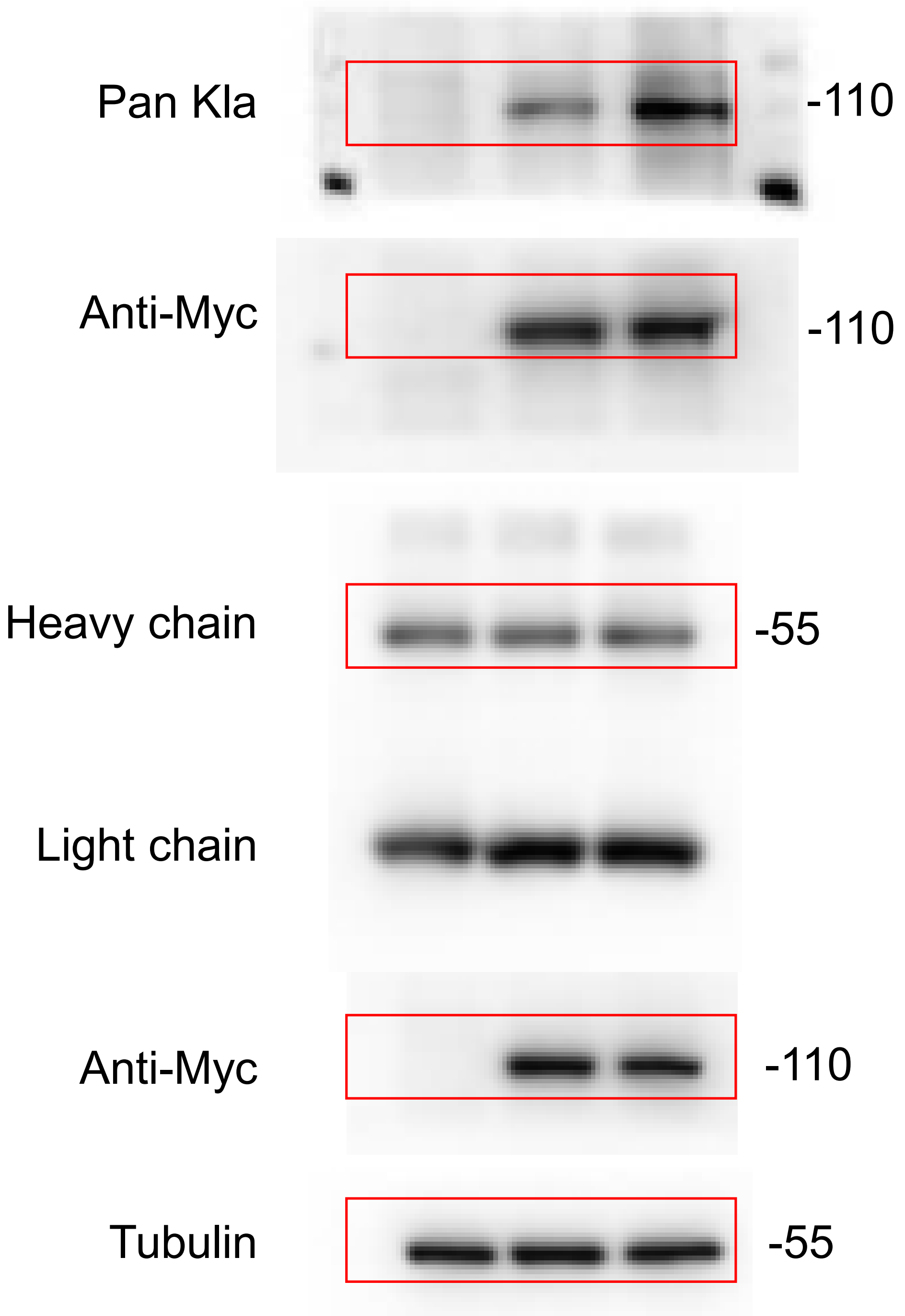
Pan Kla
-110
Anti-Myc
-110
Heavy chain
-55
Light chain
Anti-Myc
-110
Tubulin
-55

Figure 2G

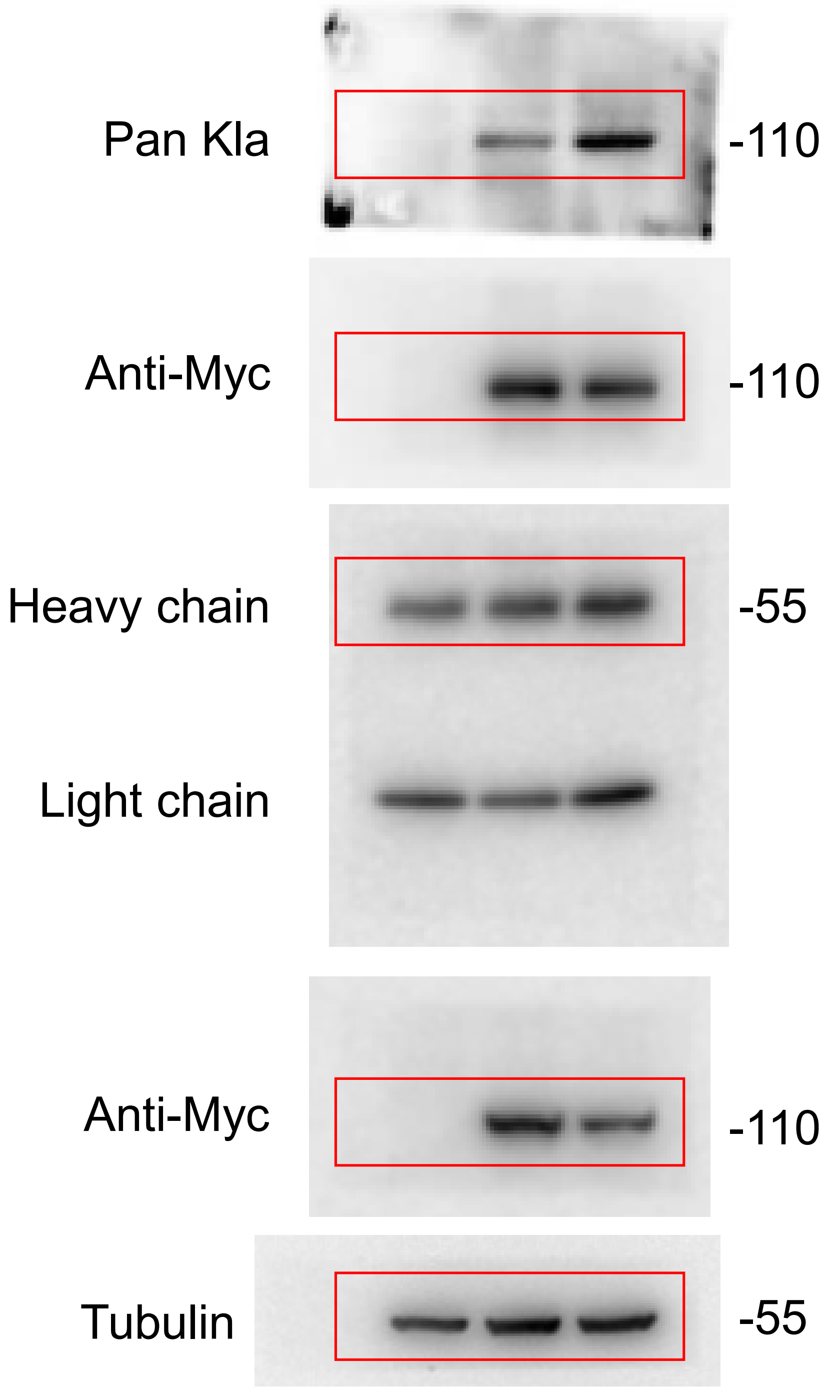

Pan Kla
-110
Anti-Myc
-110
Heavy chain
-55
Light chain
Anti-Myc
-110
Tubulin
-55

Figure 3B

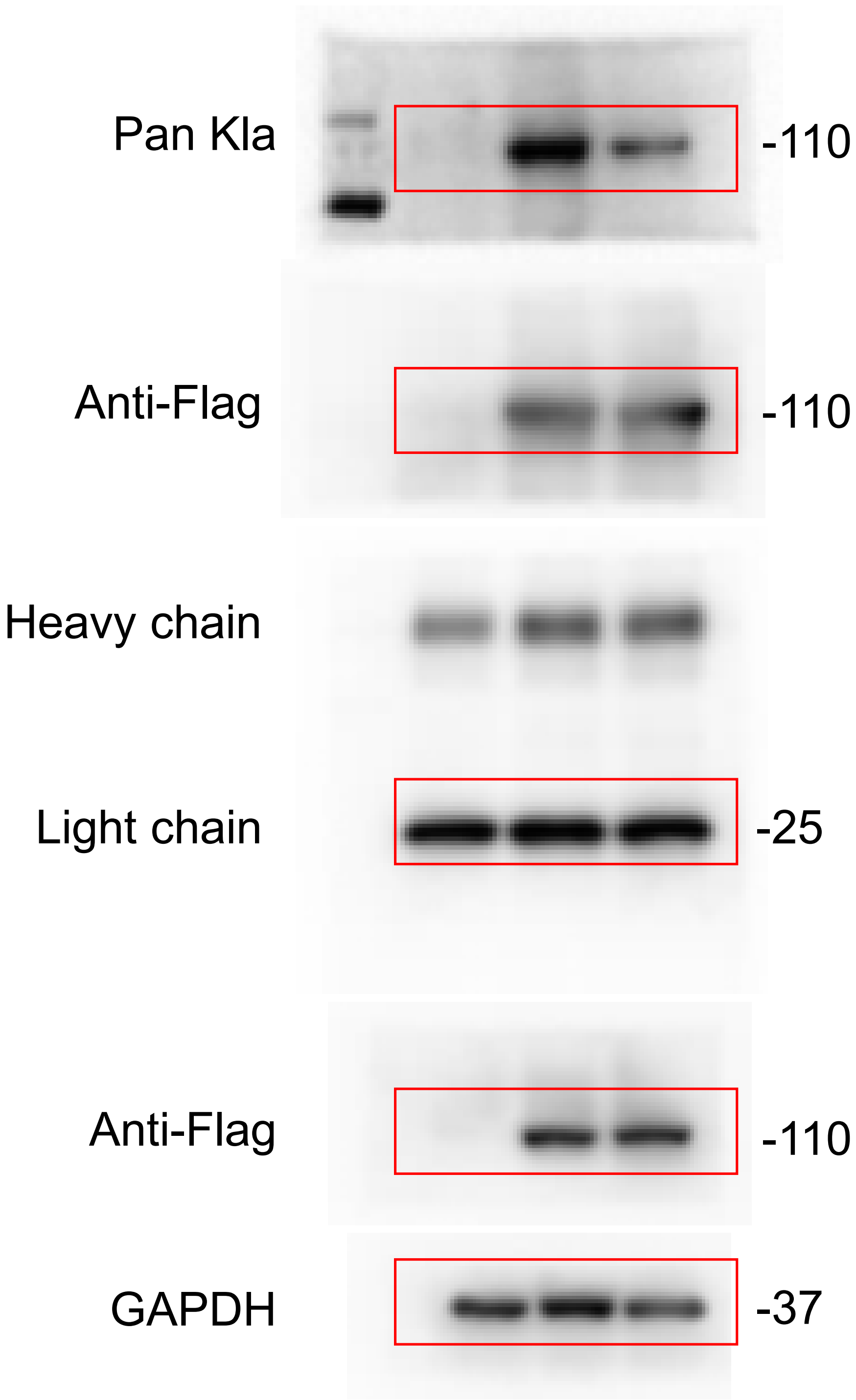

Pan Kla
-110
Anti-Flag
-110
Heavy chain
Light chain
-25
Anti-Flag
-110
GAPDH
-37

Figure 3D

ac^4C dot blot Methylene blue

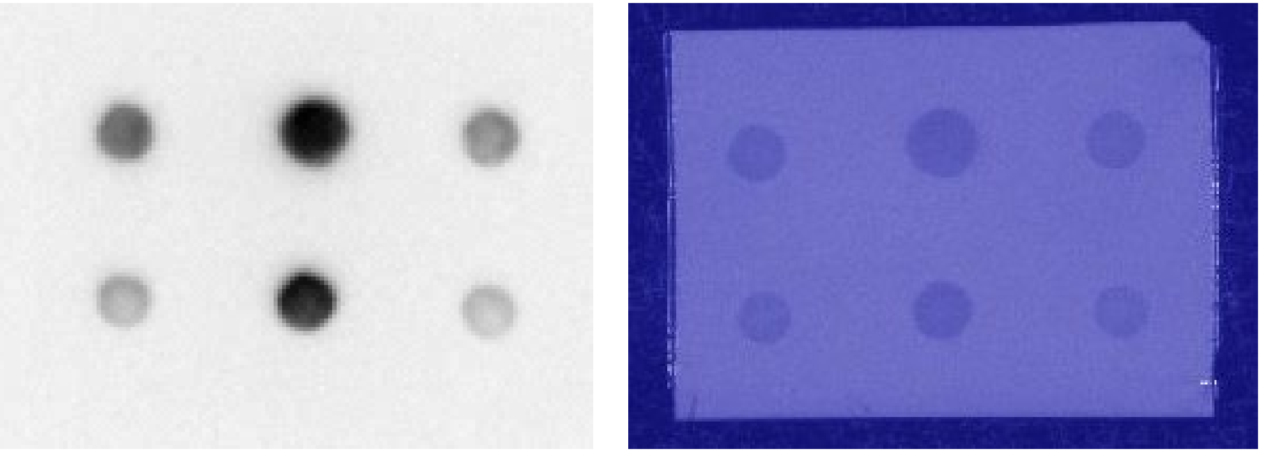

Figure 3E

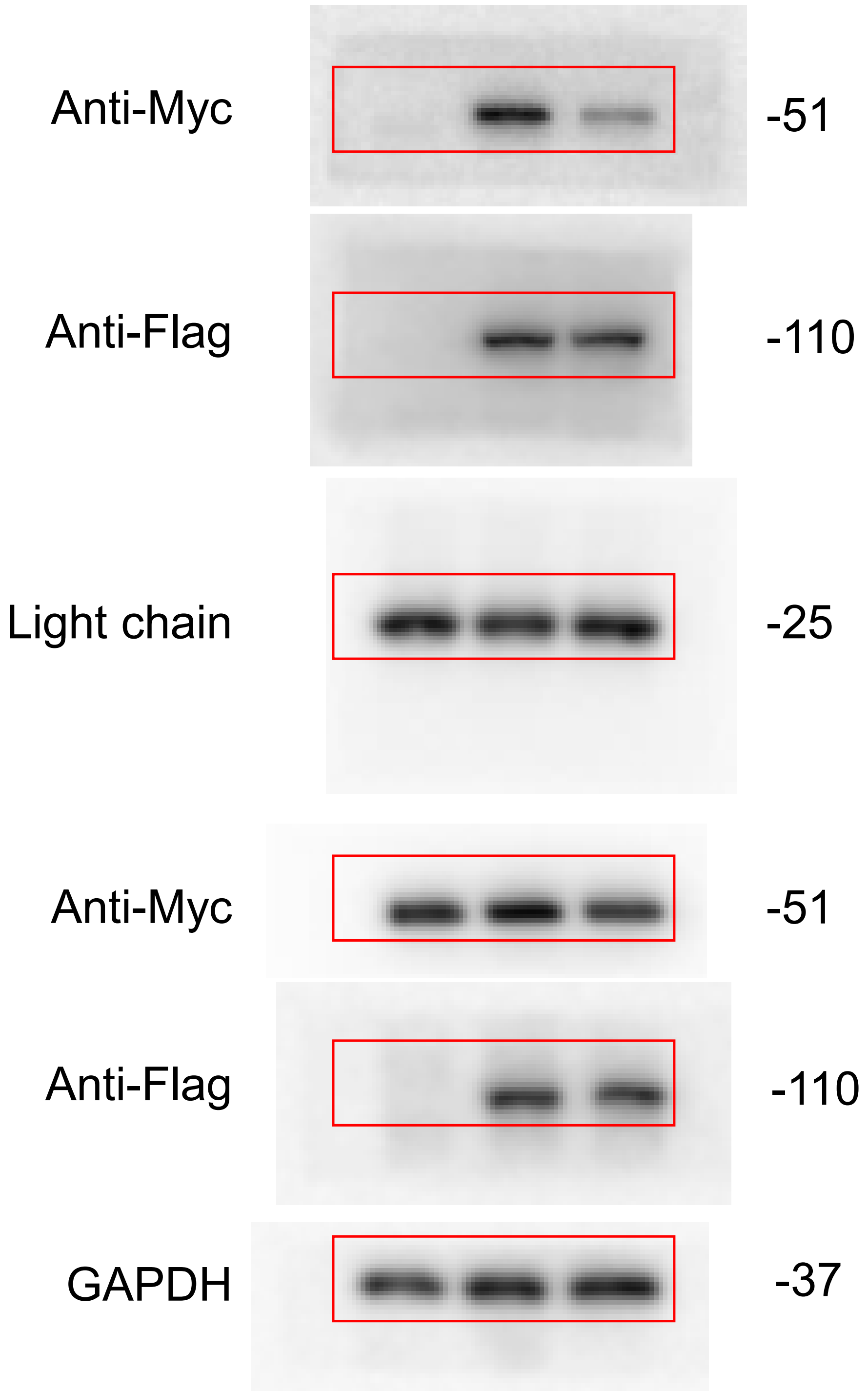
Anti-Myc
-51
Anti-Flag
-110
Light chain
-25
Anti-Myc
-51
Anti-Flag
-110
GAPDH
-37

Figure 3F

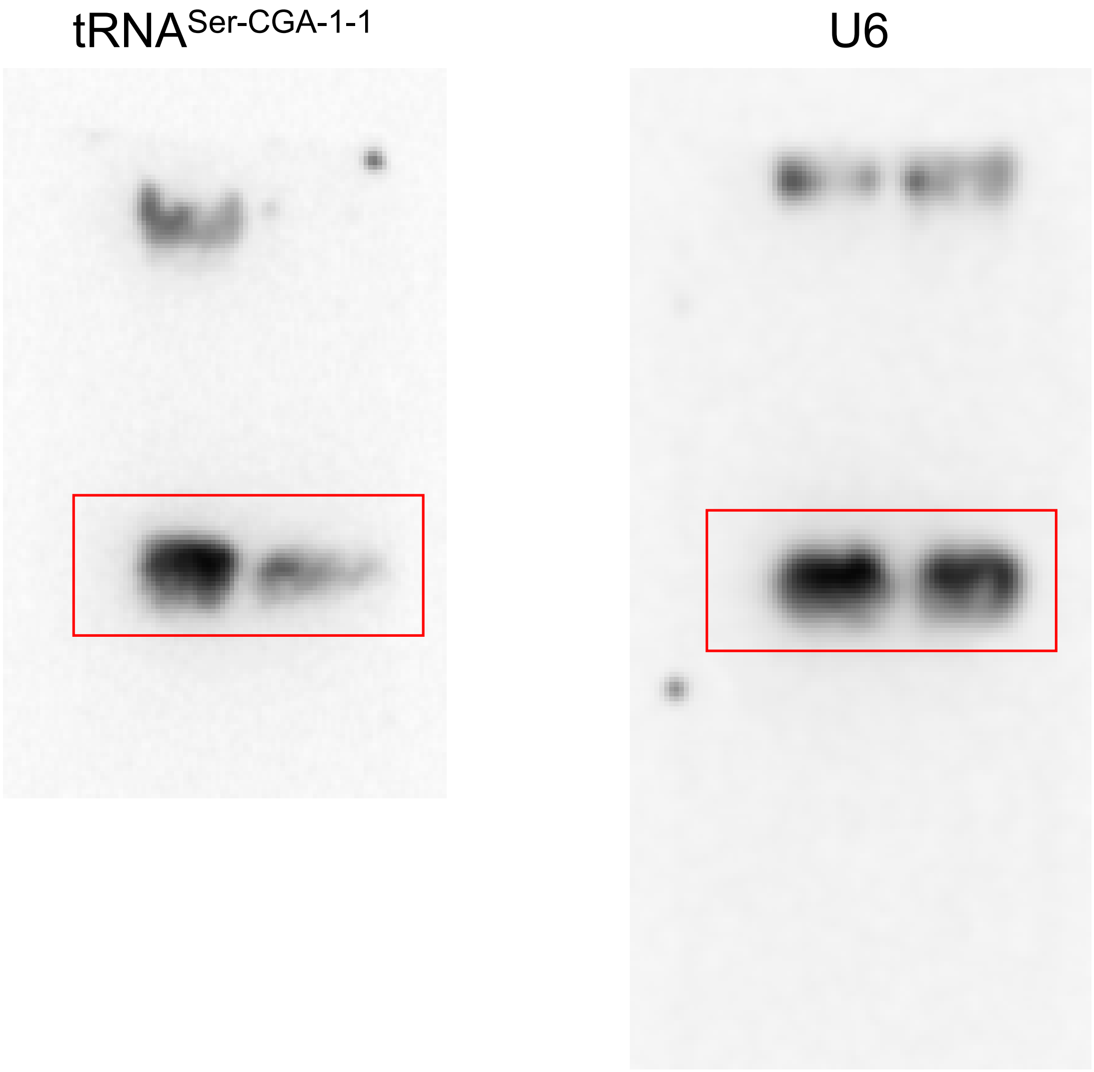

Figure 3G

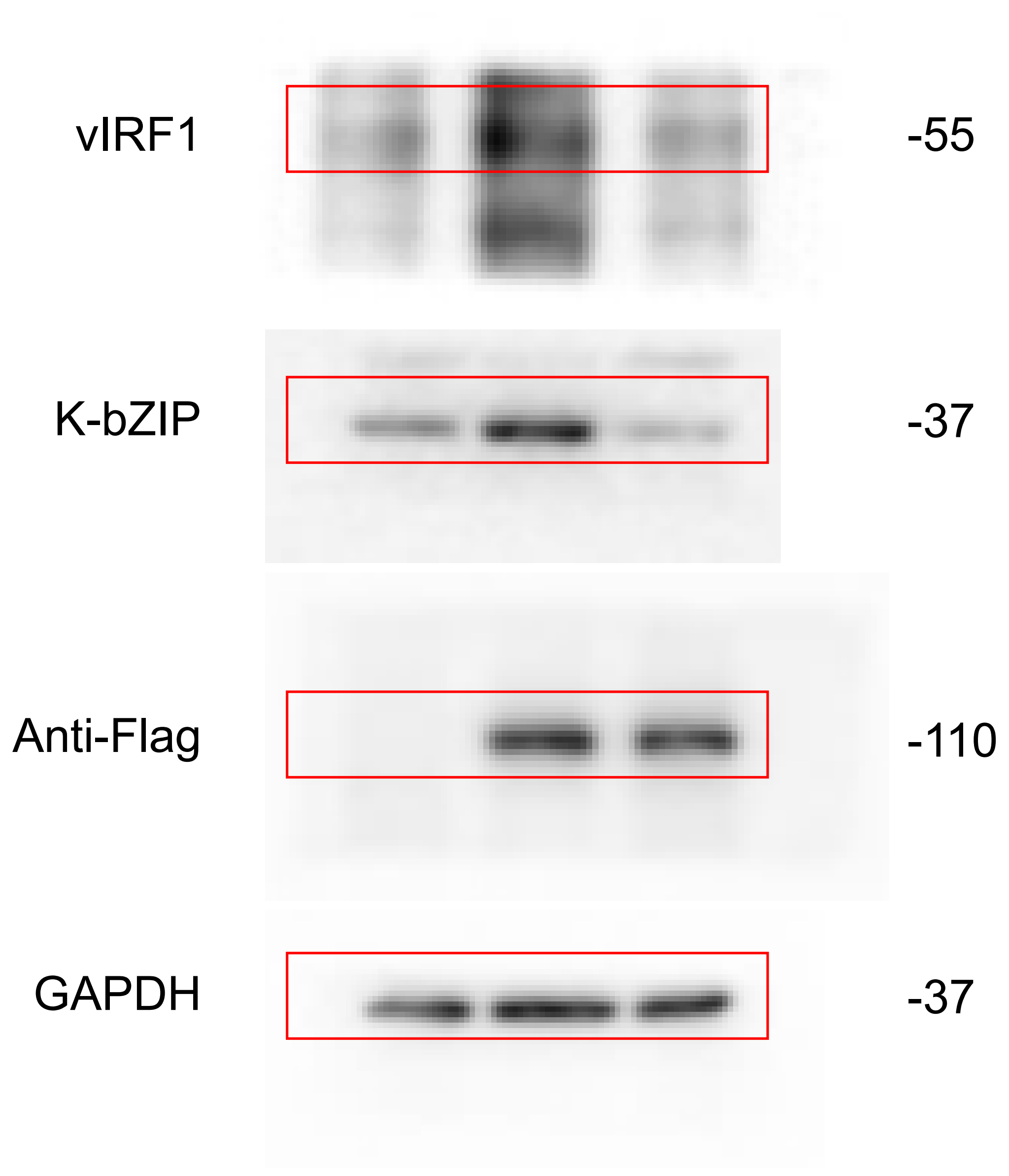

Figure 4A

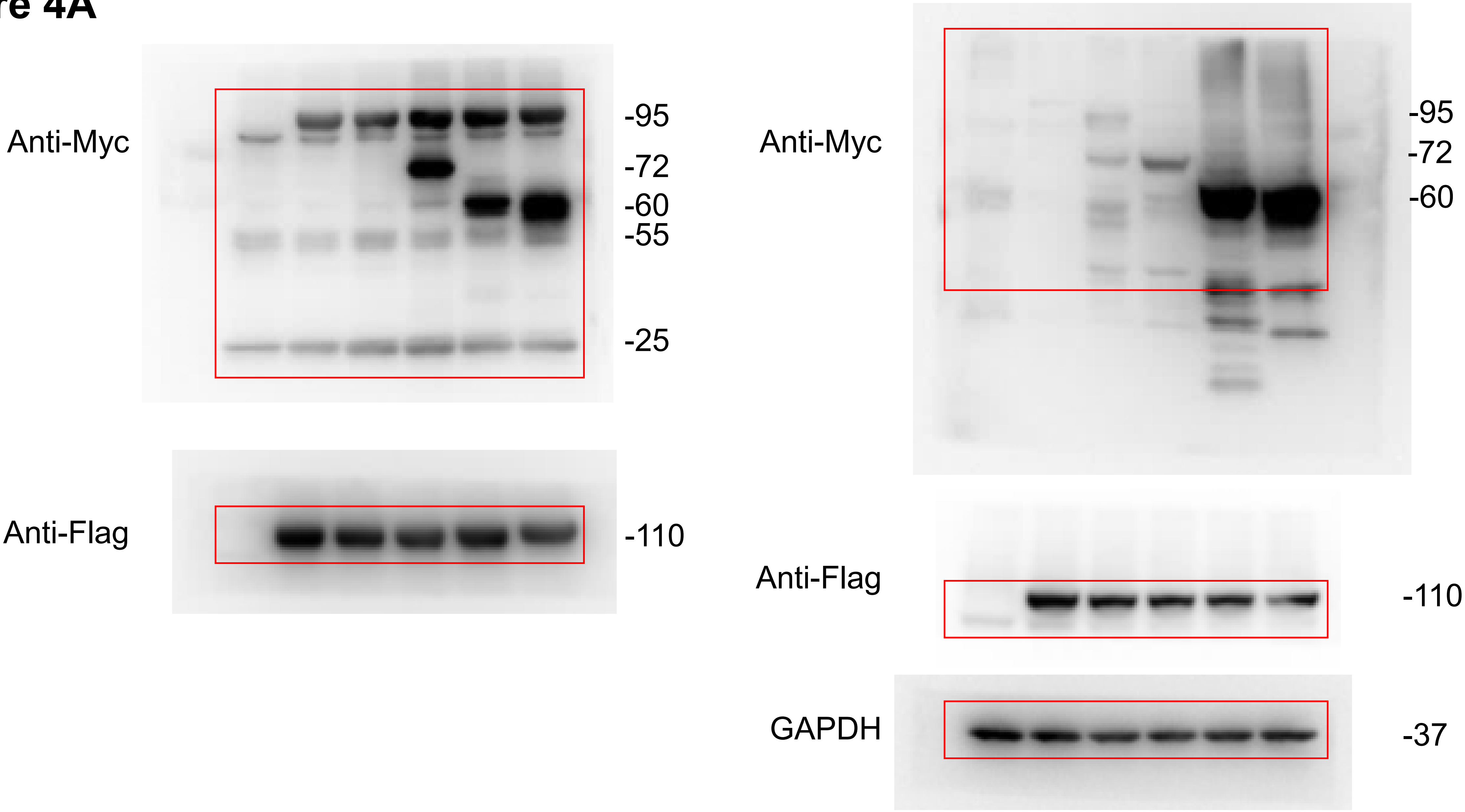
Anti-Myc
-95
-72
-60
-55
-25
Anti-Flag
-110
Anti-Myc
-95
-72
-60
Anti-Flag
-110
GAPDH
-37

Figure 4B

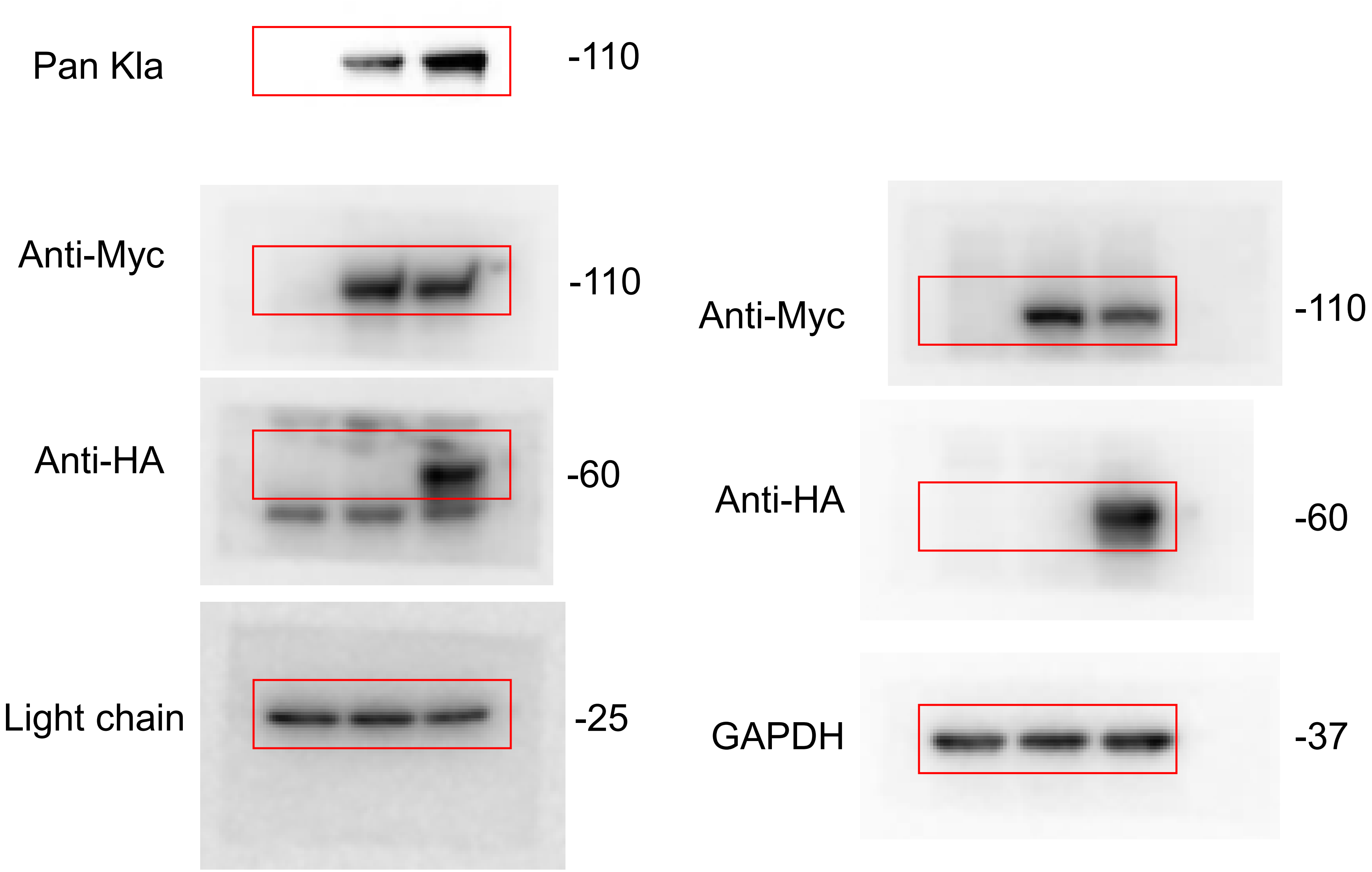
Pan Kla
-110
Anti-Myc
-110
Anti-HA
-60
Light chain
-25
Anti-Myc
-110
Anti-HA
-60
GAPDH
-37

Figure 4C

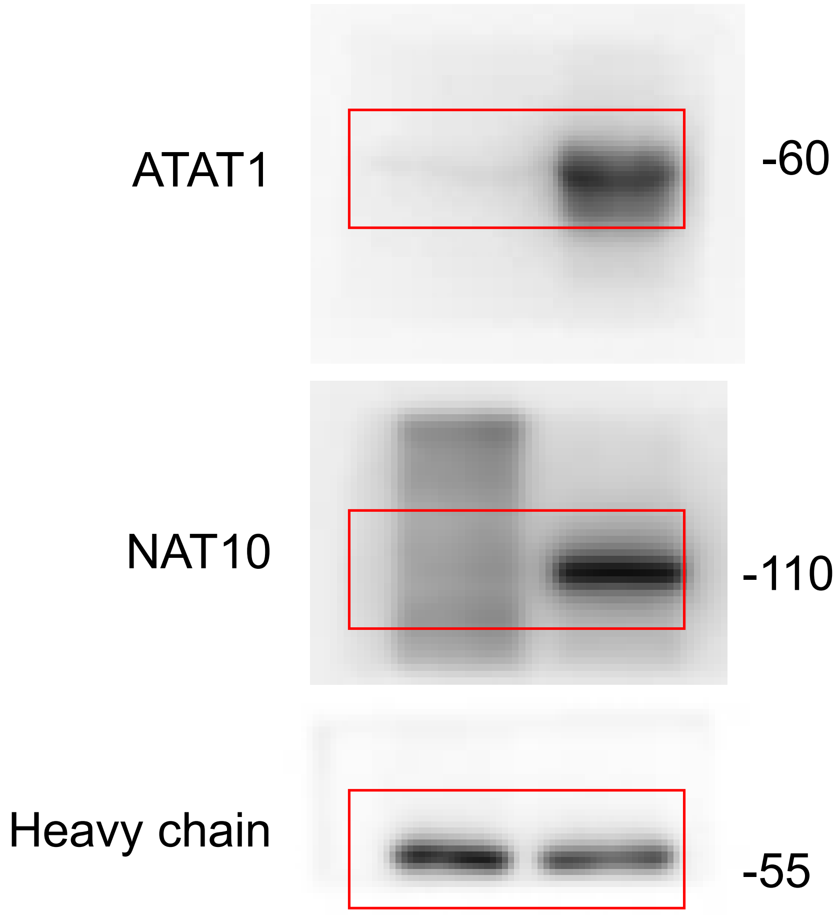

Figure 4E

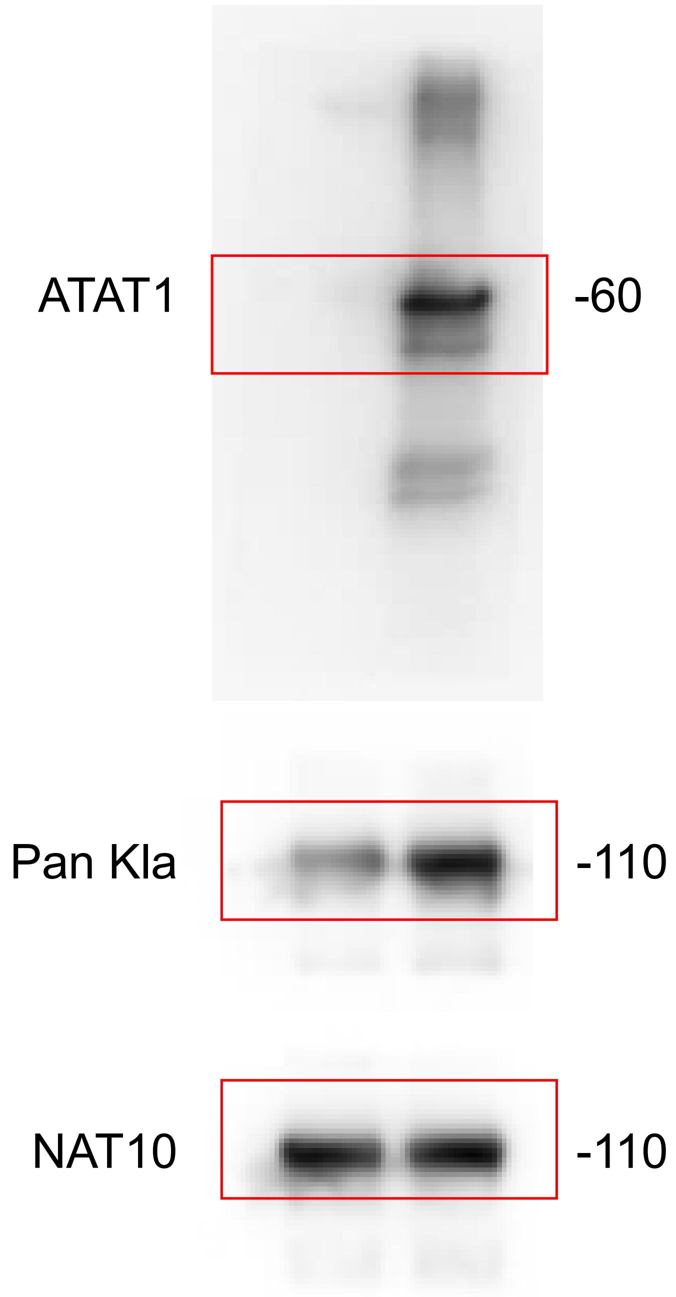

Figure 4F

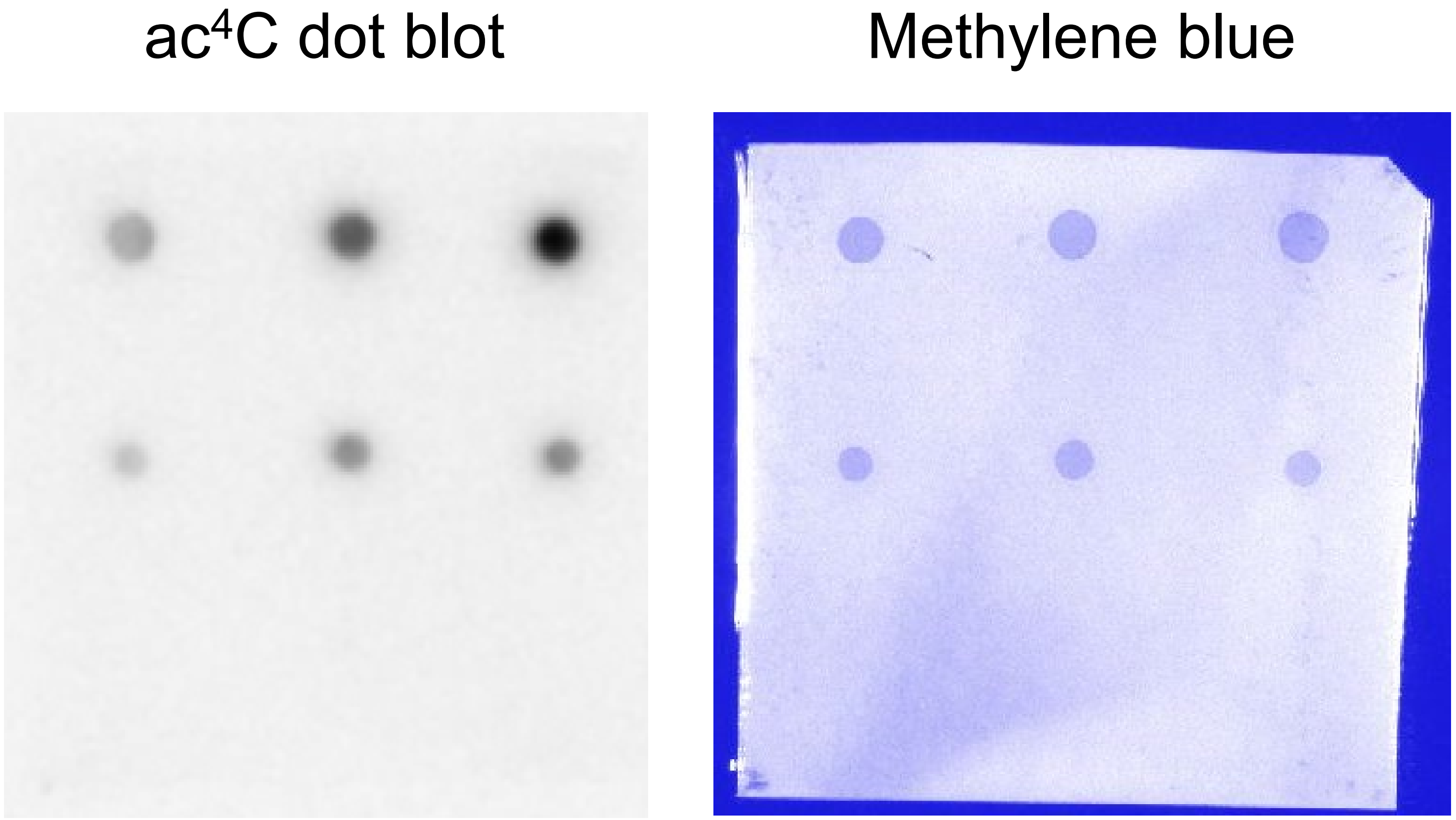

Figure 4H

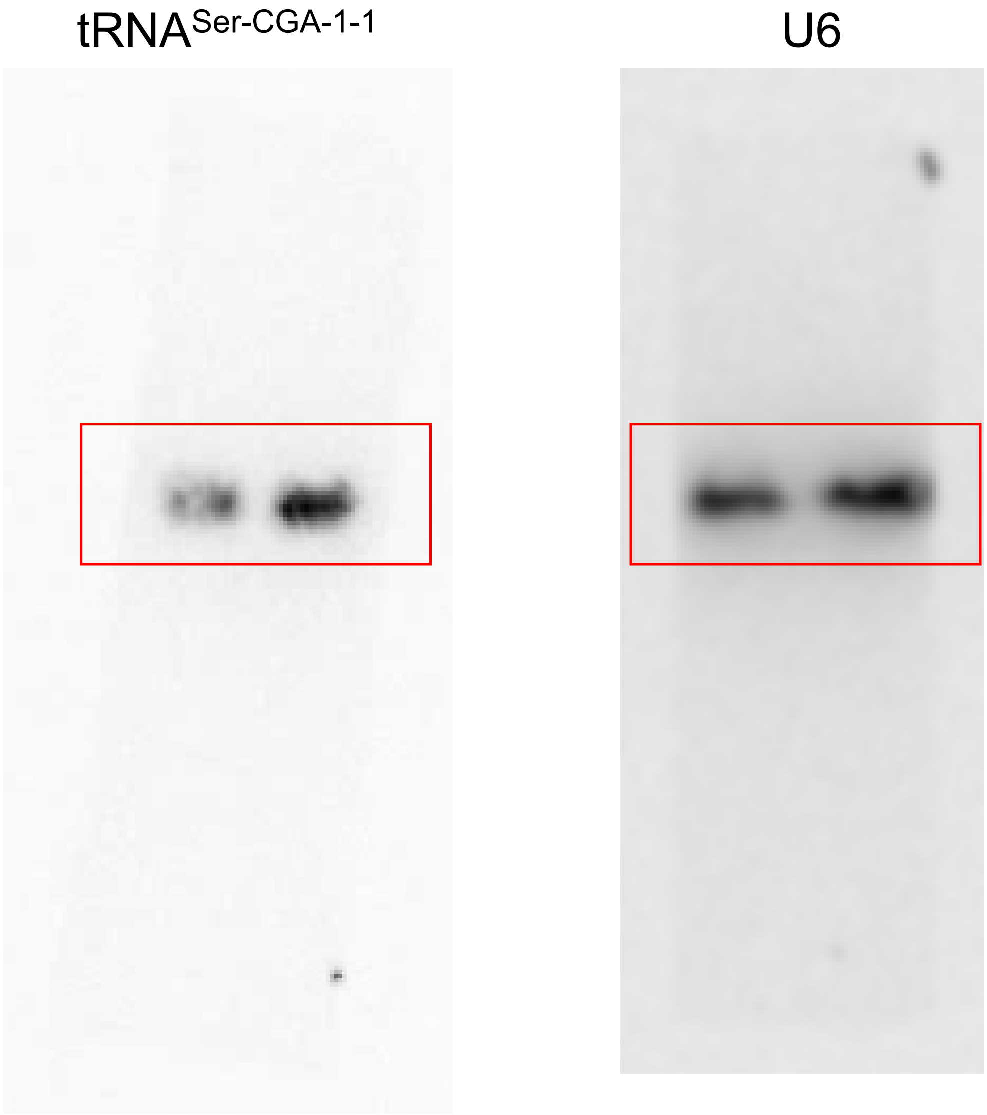

Figure 4I

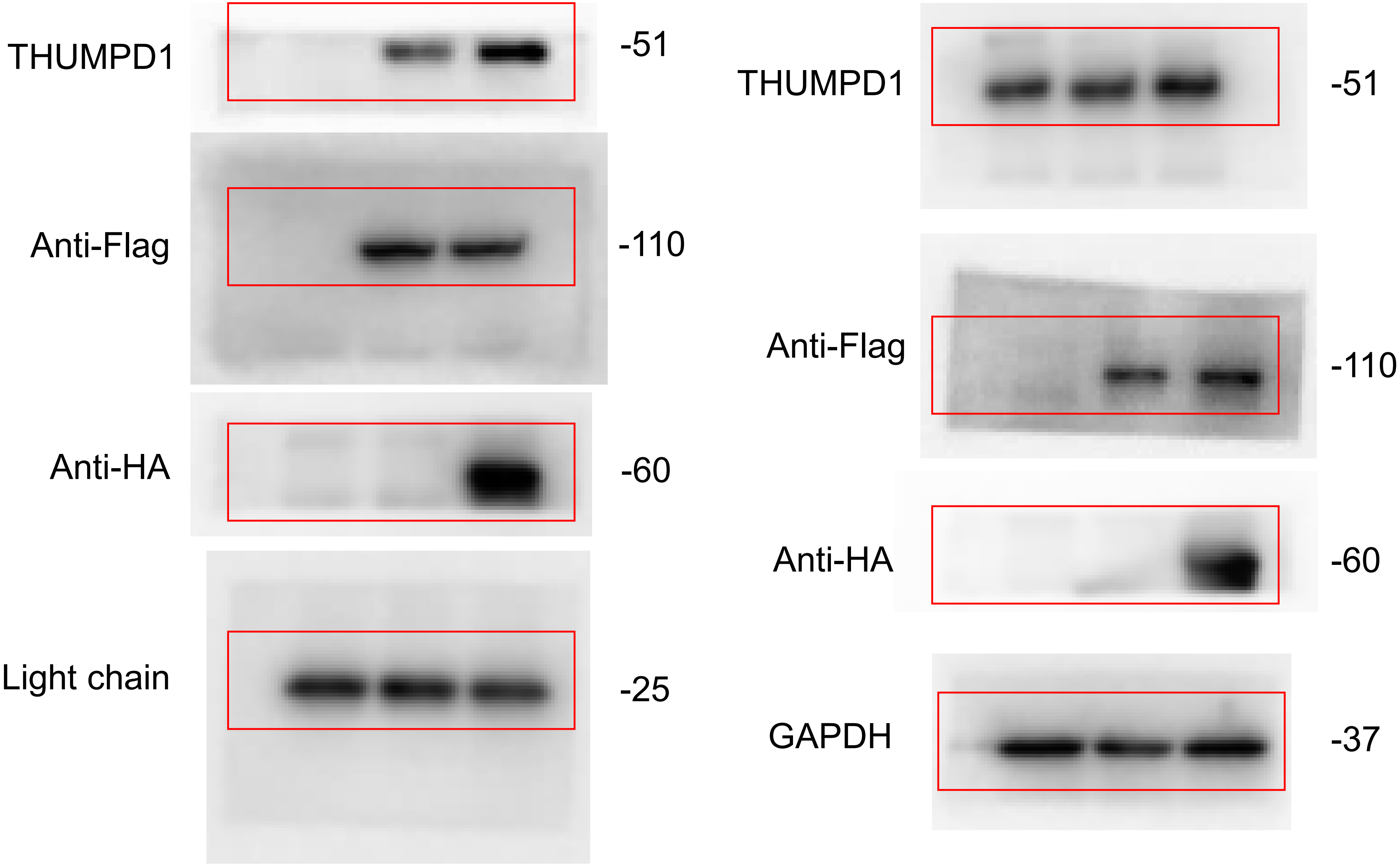
THUMPD1
-51
Anti-Flag
-110
Anti-HA
-60
Light chain
-25
THUMPD1
-51
Anti-Flag
-110
Anti-HA
-60
GAPDH
-37

Figure 5A

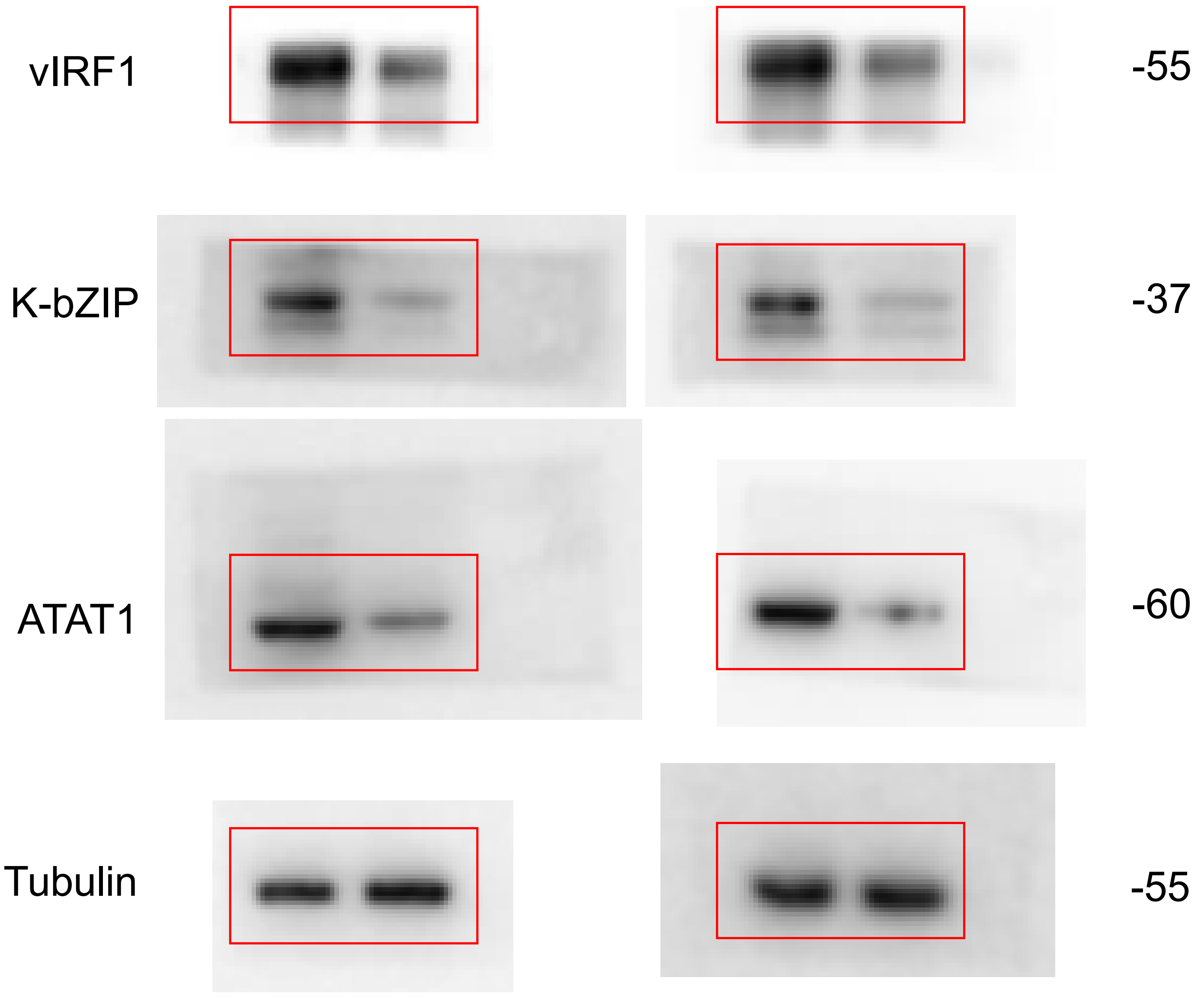

Figure 6A

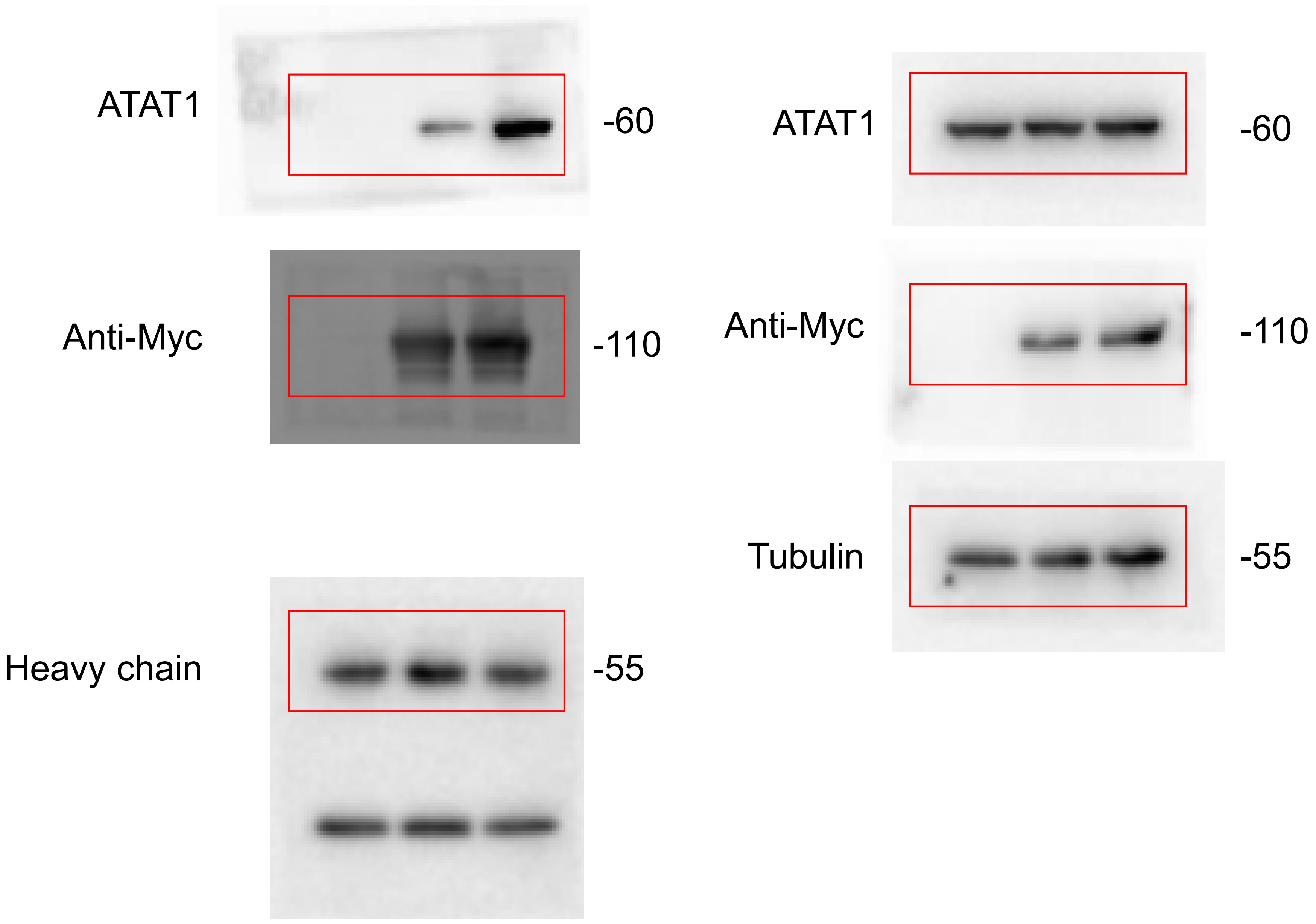
ATAT1
-60
Anti-Myc
-110
Heavy chain
-55
ATAT1
-60
Anti-Myc
-110
Tubulin
-55

Figure 6D

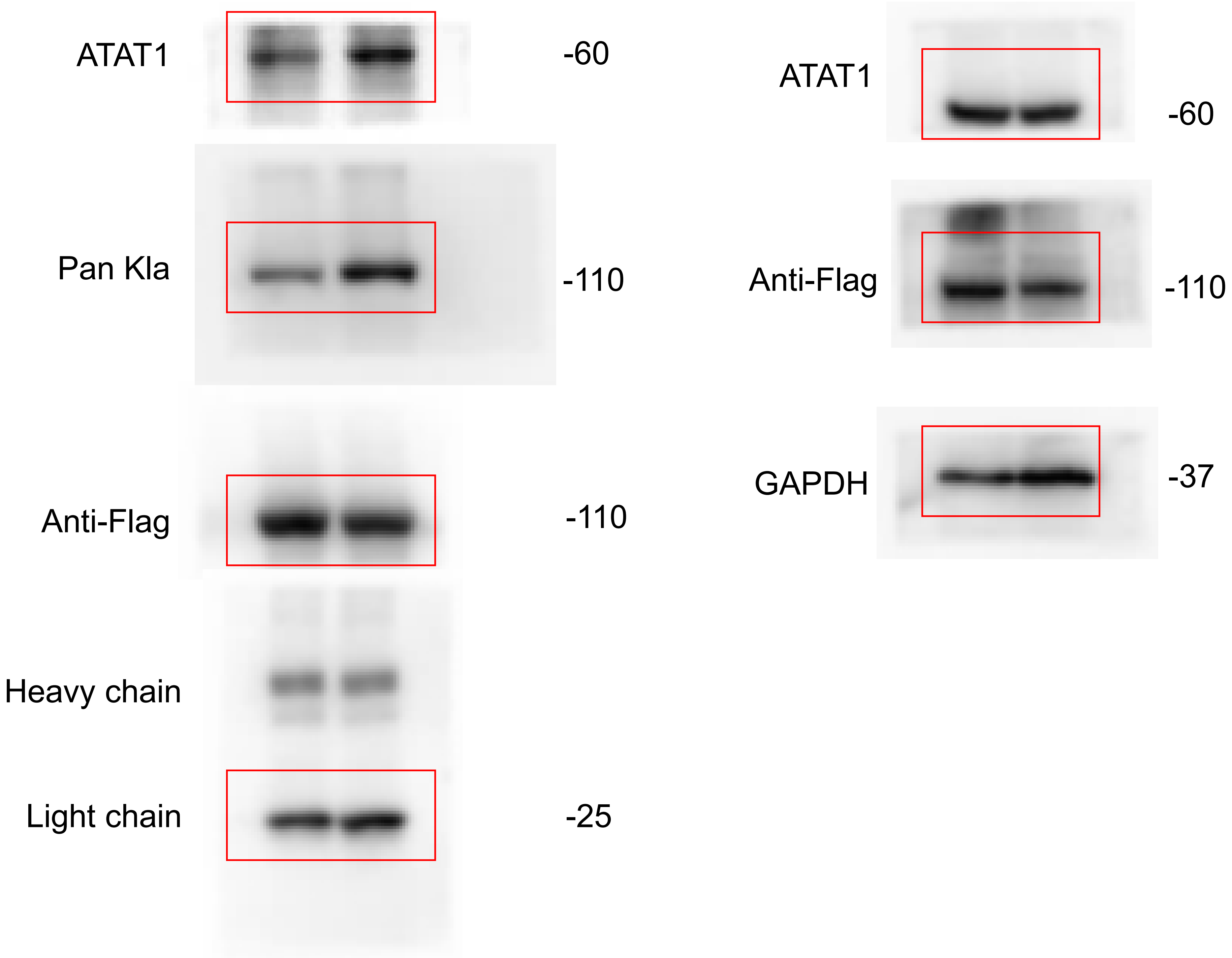
ATAT1
-60
Pan Kla
-110
Anti-Flag
-110
Heavy chain
Light chain
-25
ATAT1
-60
Anti-Flag
-110
GAPDH
-37

Figure 6E

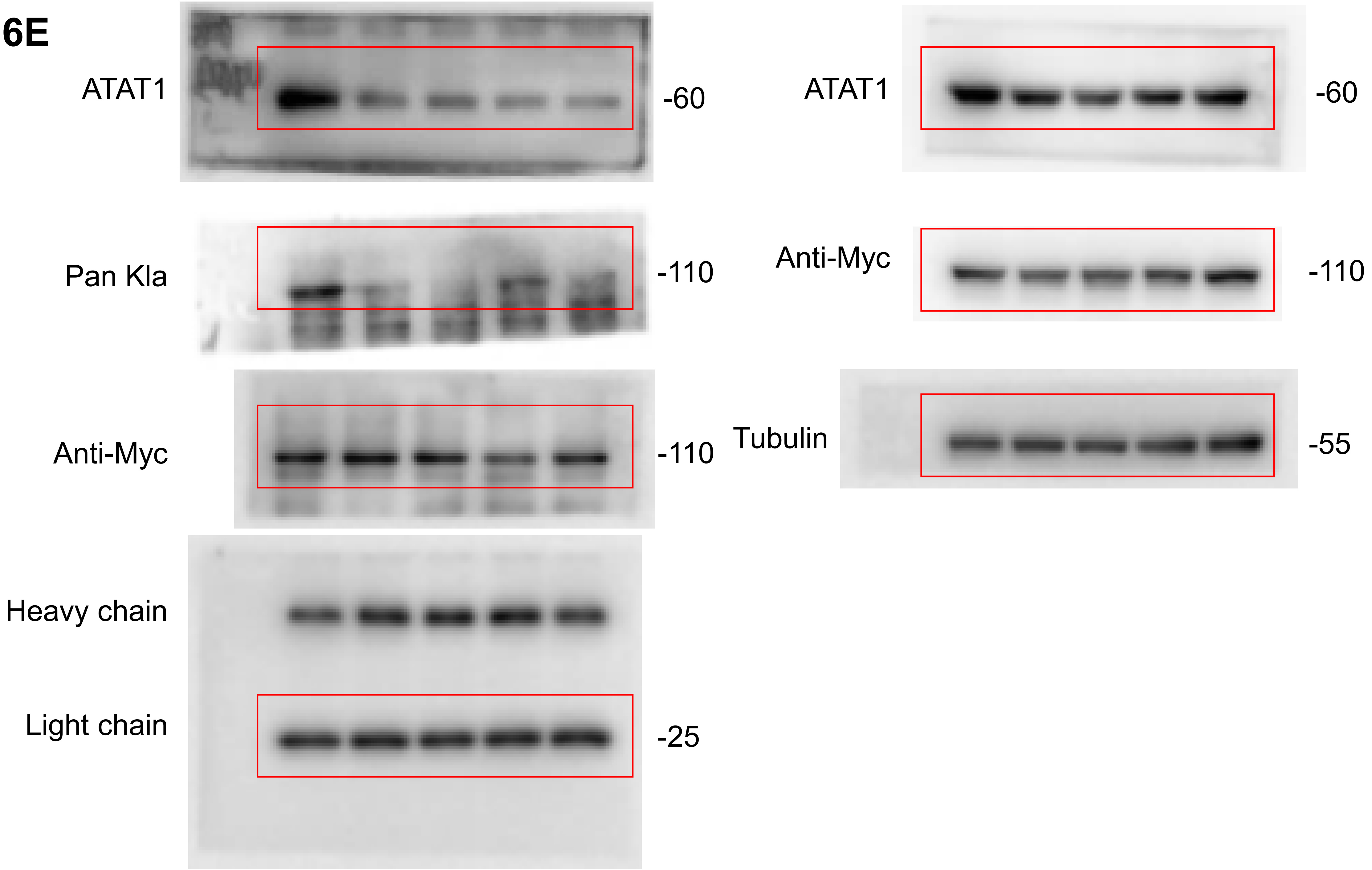
ATAT1
-60
Pan Kla
-110
Anti-Myc
-110
Heavy chain
Light chain
-25
ATAT1
-60
Anti-Myc
-110
Tubulin
-55

Figure 6F

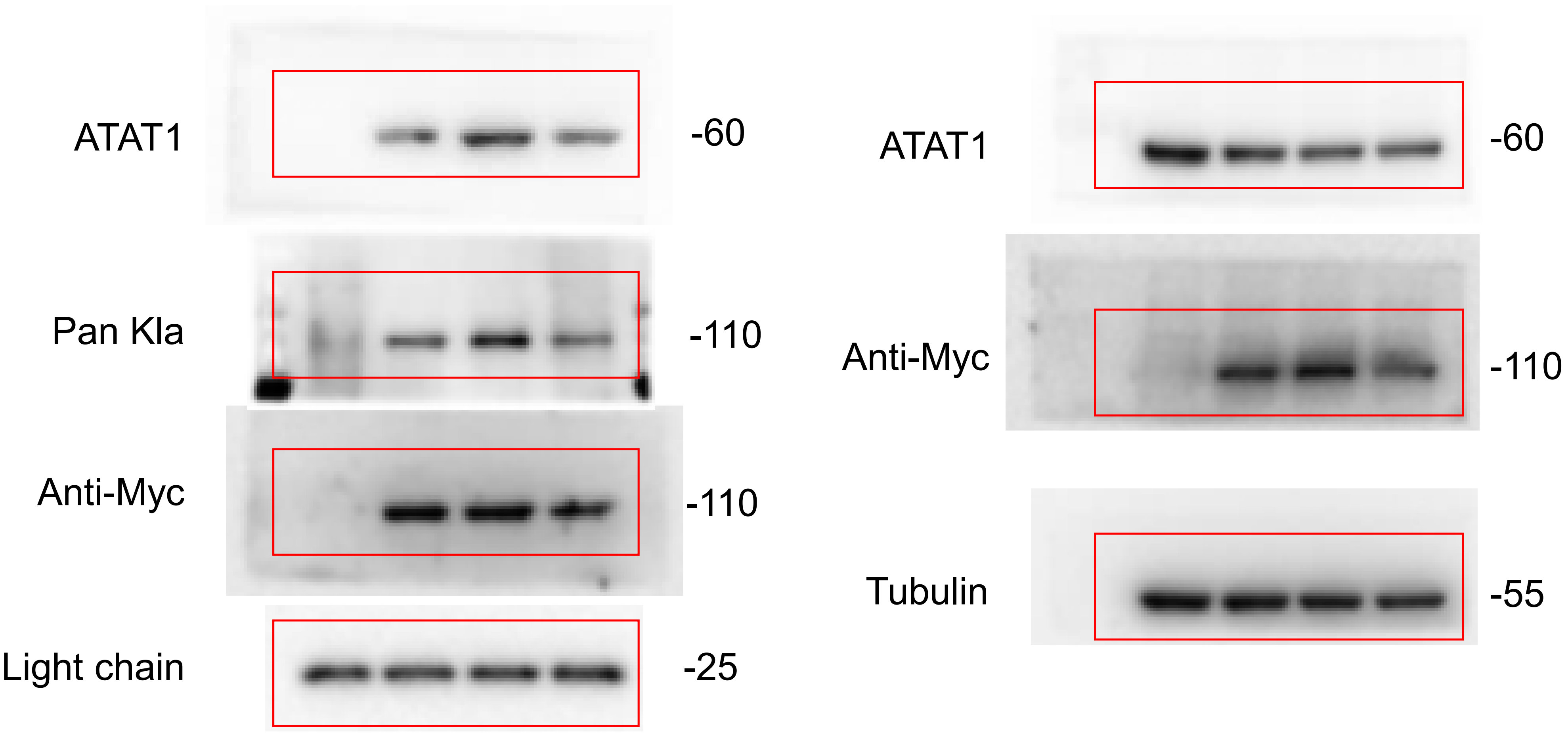

ATAT1
-60
Pan Kla
-110
Anti-Myc
-110
Light chain
-25
ATAT1
-60
Anti-Myc
-110
Tubulin
-55

Figure S2A

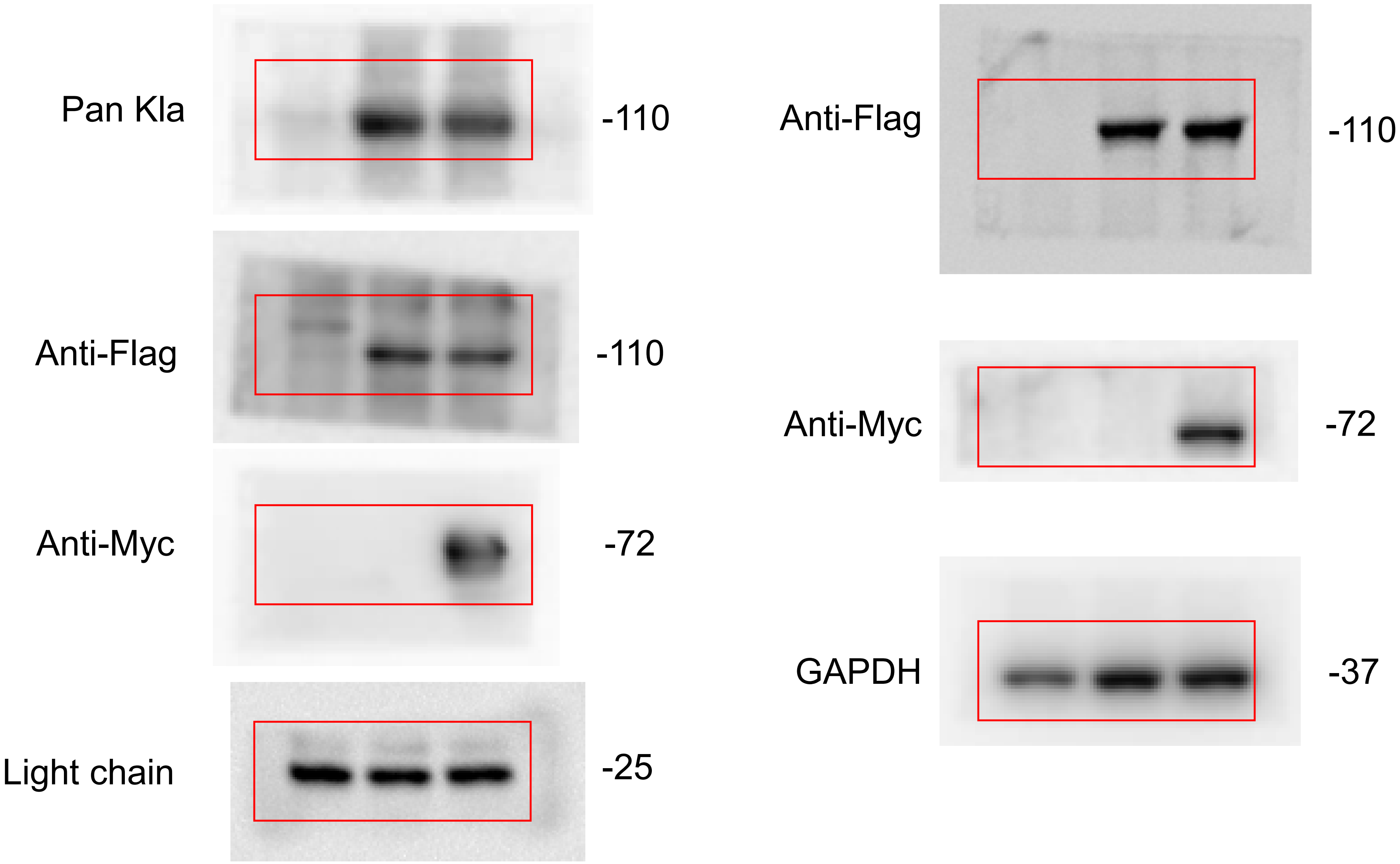

Pan Kla
-110
Anti-Flag
-110
Anti-Myc
-72
Light chain
-25
Anti-Flag
-110
Anti-Myc
-72
GAPDH
-37

Figure S2B

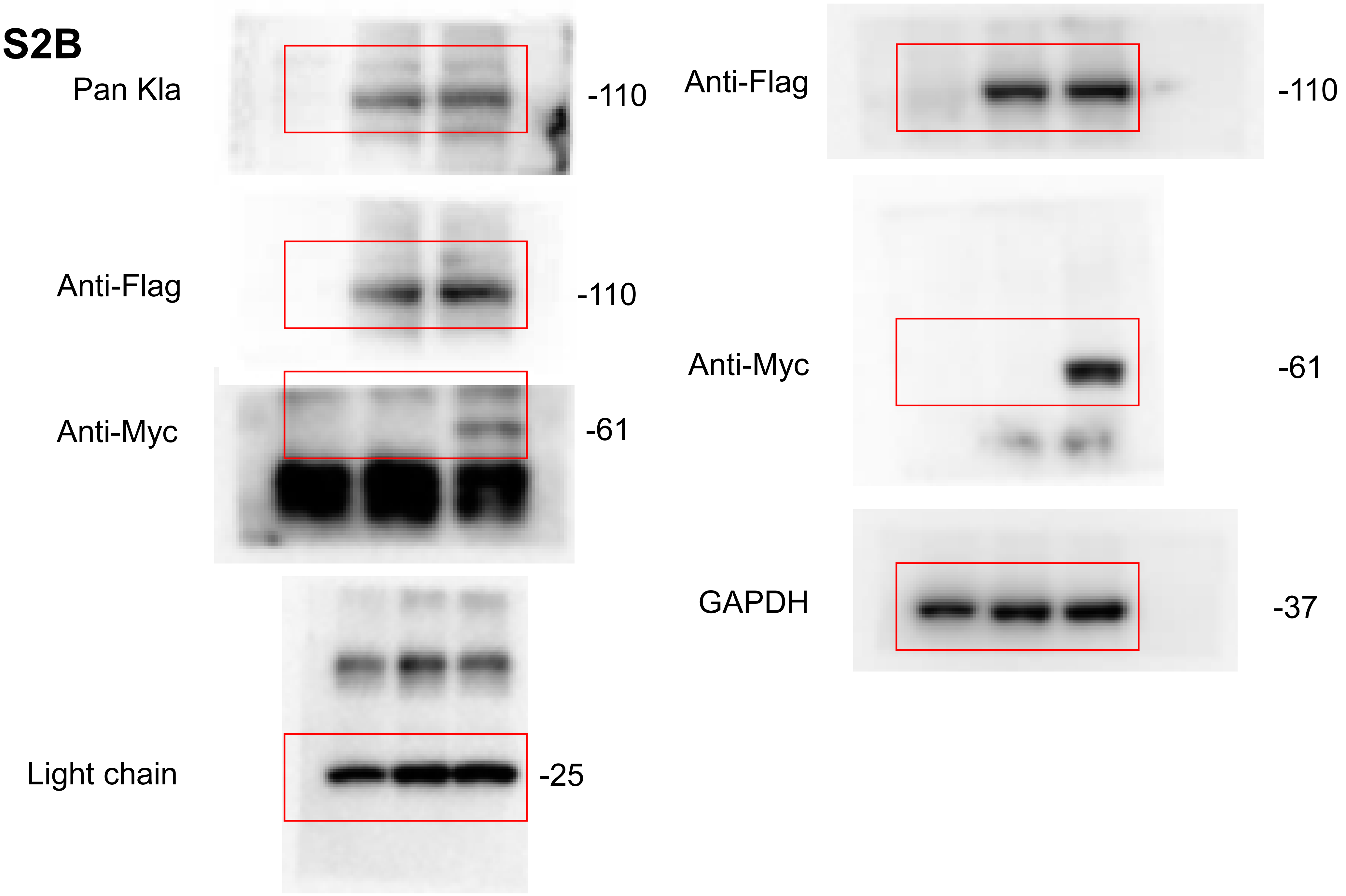

Pan Kla
-110
Anti-Flag
-110
Anti-Myc
-61
Light chain
-25
Anti-Flag
-110
Anti-Myc
-61
GAPDH
-37

Figure S3

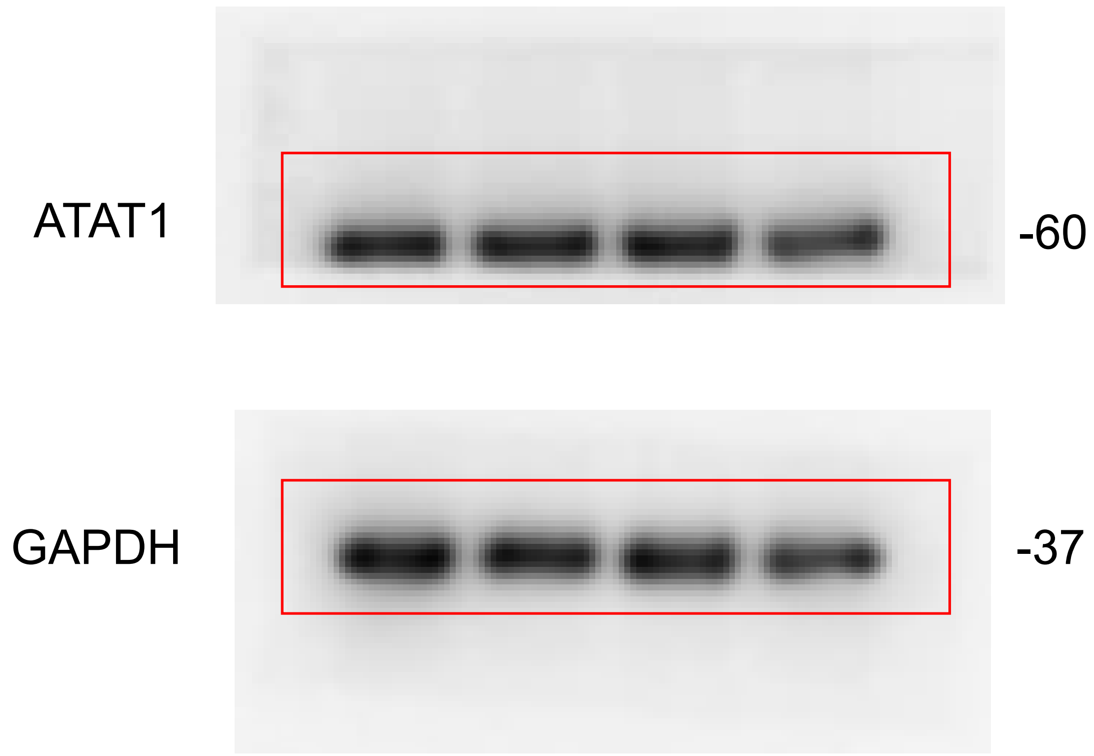

Figure S4

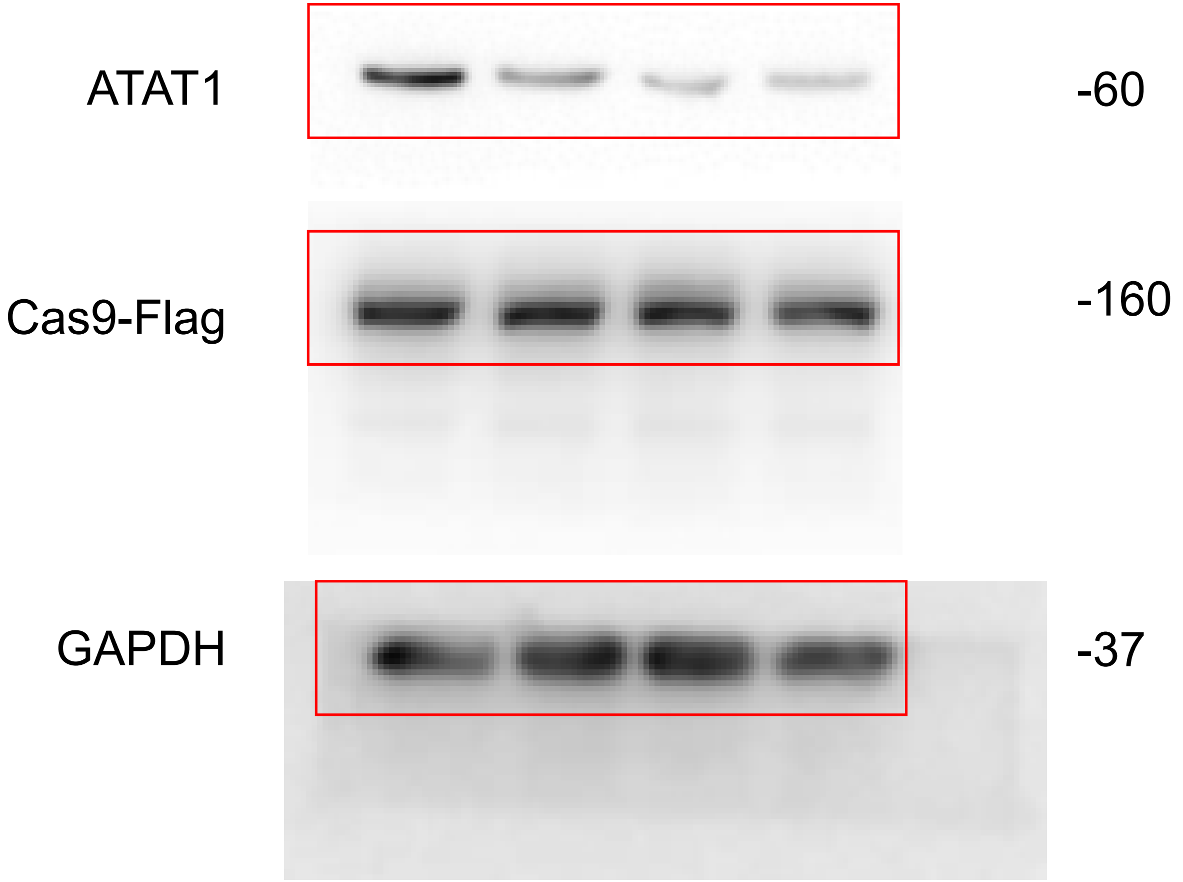

Supplement: Supplementary file 8 — Original data of WB, NB and Dot blot [file 41418_2024_1327_MOESM8_ESM.pdf]
